# Mobility of *mPing* and its associated elements is regulated by both internal and terminal sequences

**DOI:** 10.1186/s13100-023-00289-3

**Published:** 2023-02-11

**Authors:** Priscilla S. Redd, Stephanie Diaz, David Weidner, Jazmine Benjamin, C. Nathan Hancock

**Affiliations:** 1 Department of Biology and Geology, University of South Carolina Aiken, Aiken, SC 29801 USA; 2 Present address: Bayer Pharmaceuticals, Broad Institute of MIT and Harvard, Cambridge, MA 02142 USA; 3 Present address: Division of Nephrology, Department of Medicine, University of Alabama at Birmingham, Birmingham, AL 35233 USA

**Keywords:** *mPing*, Transposition complex, Terminal inverted repeats

## Abstract

**Background:**

DNA transposable elements are mobilized by a “cut and paste” mechanism catalyzed by the binding of one or more transposase proteins to terminal inverted repeats (TIRs) to form a transpositional complex. Study of the rice genome indicates that the *mPing* element has experienced a recent burst in transposition compared to the closely related *Ping* and *Pong* elements. A previously developed yeast transposition assay allowed us to probe the role of both internal and terminal sequences in the mobilization of these elements.

**Results:**

We observed that *mPing* and a synthetic *mPong* element have significantly higher transposition efficiency than the related autonomous *Ping* and *Pong* elements. Systematic mutation of the internal sequences of both *mPing* and *mPong* identified multiple regions that promote or inhibit transposition. Simultaneous alteration of single bases on both *mPing* TIRs resulted in a significant reduction in transposition frequency, indicating that each base plays a role in efficient transposase binding. Testing chimeric *mPing* and *mPong* elements verified the important role of both the TIRs and internal regulatory regions*.* Previous experiments showed that the G at position 16, adjacent to the 5′ TIR, allows m*Ping* to have higher mobility. Alteration of the 16th and 17th base from *mPing’s* 3′ end or replacement of the 3′ end with *Pong* 3′ sequences significantly increased transposition frequency.

**Conclusions:**

As the transposase proteins were consistent throughout this study, we conclude that the observed transposition differences are due to the element sequences. The presence of sub-optimal internal regions and TIR bases supports a model in which transposable elements self-limit their activity to prevent host damage and detection by host regulatory mechanisms. Knowing the role of the TIRs, adjacent sub-TIRs, and internal regulatory sequences allows for the creation of hyperactive elements.

**Supplementary Information:**

The online version contains supplementary material available at 10.1186/s13100-023-00289-3.

## Background

Transposable elements (TEs) are mobile segments of DNA present in essentially all eukaryotic genomes. They are particularly prevalent in plant genomes, which often contain a large percentage of TE-derived DNA sequences [1–3]. One of the most active transposable elements in the rice genome is *mPing*, a 430 base pair deletion derivative of the larger *Ping* element [4–6]. *mPing* belongs to the *PIF/Harbinger* superfamily of TEs and is classified as a miniature inverted repeat TE (MITE) because of its small size, reliance on other elements for mobilization, and abundance in the genome [7–9]. Because this element is still active, we can test its transposition mechanisms and make inferences about the functional significance of these elements on the genome.

*mPing* and its related elements are part of the larger category of DNA elements (Class II) that have short reverse complementary sequences known as terminal inverted repeat (TIR) sequences on both ends of the element. These TIR sequences are bound by Transposase (TPase) proteins which catalyze transposition. TPase proteins are characterized by a catalytic DDE/D amino acid triad required for DNA cleavage [10]. The formation of an active transposition complex between the element TIRs and the TPase proteins is a key step in mobilizing DNA TEs [11, 12]. Unlike other TE superfamilies, the *PIF/Harbinger* elements require two proteins: ORF1, for DNA binding and the DDE/D containing TPase for mobilization [13–15]. We hypothesize that the ORF1, TPase, and TIR sequences interact to form a complex that facilitates transposition.

Analysis of *mPing, Ping,* and *Pong* element copy numbers in over 3000 domesticated and wild rice genomes provides a window into the recent burst in *mPing* activity during rice domestication [16]. *mPing* has a relatively high copy number, with an average of 9.2 copies in domesticated cultivars. In contrast, the autonomous *Ping* and *Pong* elements have 0.09 and 4.25 copies on average [16]. Based on this observation, we hypothesize that the low copy number of *Ping* may allow it to evade host silencing mechanisms. Thus, the unique combination of ORF1 and TPase proteins expressed from the relatively low copy *Ping* element combined with a compatible hyperactive MITE makes it possible for the transposition burst. In contrast, *Pong* appears to be highly regulated epigenetically [17] and is thought only to mobilize *mPing* during tissue culture when DNA methylation decreases [4, 18]. While no naturally occurring non-autonomous MITE of *Pong* exists, synthetic versions of *mPong* can be mobilized in yeast [14]. We used these MITEs to identify the sequences required for transposition, allowing us to further understand the transposition mechanism of this important TE superfamily.

The TIRs of *mPing, Ping,* and *Pong* follow the conserved pattern observed for elements in the *PIF/Harbinger* superfamily [19]. This high degree of sequence conservation is consistent with the prediction that TIRs act as the region where TPase and ORF1 proteins bind and recognize the *mPing* element. Experiments with other TEs have shown that altering the TIRs or adjacent sequences (sub-TIRs) disrupts transposition. For example, deletion of the terminal 23 base pairs (bp) from the 3′ TIR of the *P-element* completely inhibited transposition [20]. Similarly, various alterations of the 12 bases on the left end of the *Sleeping Beauty* element significantly disrupted its transposition [21], and mutation of the four terminal bases of the *Tc3* element almost completely abolished transposition [22]. Detailed analysis of the bacterial *Tn10* element showed that mutation for bases 1-3 and 6-13 of the TIR significantly reduced the transposition frequency [23, 24]. Simultaneous mutation of both *mPing’s* TIRs also showed that most of the terminal 14 bases are critical for transposition [16], suggesting that these highly conserved bases are directly involved in transposition complex formation. However, these experiments did not investigate if one TIR is more critical for transposition.

Previous studies have shown that sequences internal to the TIRs can also play a significant role in transposition. For example, it was shown that the rice MITE *Stowaway 35* (*Ost35*) has internal regions that promote and inhibit its transposition [25]. Similarly, the autonomous *piggyBac* element from the *Autographa californica* moth was shown to require over 1000 bp of internal sequences to achieve efficient transposition [26]. Sequences internal to the autonomous *P-element* TIR were also found to be essential for transposition [20]. These internal sequences may function by interacting with the mobilization proteins to facilitate transposition complex formation. Binding assays performed with the autonomous *Sleeping Beauty* element showed that the transposase protein preferentially binds to the direct repeat sequences that are internal to the TIRs [21], potentially initiating transposition complex formation. We hypothesized that *mPing* might also have internal sequences that could promote or inhibit transposition and contribute to its overall burst in transposition.

To address these questions, we performed systemic mutations of the *mPing* element and a synthetic 430 bp *mPong* element. We have identified internal sequence mutations and TIR mutations that directly affect their transposition, indicating these regions’ role in element regulation. We also synthesized several hybrid elements containing regions from both *mPing* and *mPong*. The results from these domain swapping assays correlate with the determined transposition promoting and repressing regions. Hyperactive elements were created by combining complementary systemic and TIR mutations. Together, these results suggest that though the *mPing* element has exhibited a burst of transposition in the rice genome, it still retains sequences designed to inhibit hyperactive transposition that could be detrimental to host survival.

## Results

### Transposition frequency of autonomous elements and their associated MITES

**Fig. 1:**
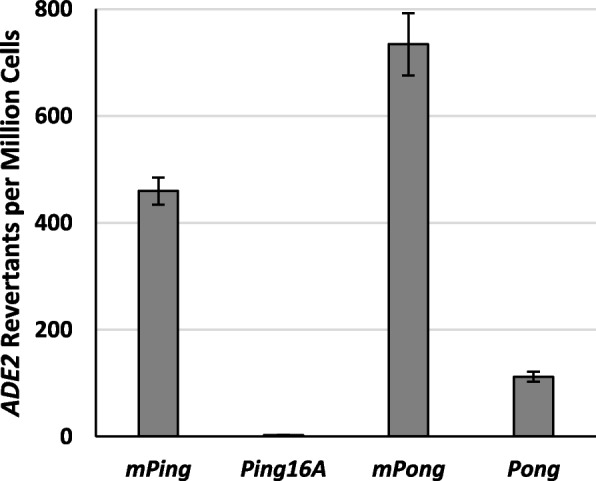
Yeast transposition rates for *mPing*, *Ping16A*, *mPong*, and *Pong*. Columns represent the average and error bars represent standard error (*n* = 6)

We tested the transposition frequency of the *mPing*, *Ping16A* (the version associated with *mPing* bursts)*, mPong,* and *Pong* elements using the previously established yeast transposition assay and high activity transposase proteins [ORF1 Shuffle 1 and *Pong* TPase L418/420A] [14, 27]. These versions of the ORF1 and TPase proteins have been optimized for transposition in yeast by strengthening the nuclear localization signal and removing the nuclear export signal. In this assay, element excision from the *ADE2* gene and precise repair of the excision site determines the frequency of colonies that can grow on plates lacking adenine. We observed that the *mPing* and *mPong* elements transposed at significantly higher frequencies (*p* < 0.05) than *Ping16A* and *Pong*, with *Ping16A* transposition appearing to be lower than *Pong* transposition (Fig. 1). The relative element activity appears to correlate with the previously observed relative abundance of the natural elements in rice genomes [16]. These results suggest that the overall element copy number in a species is partially attributed to element mobility. In addition, this result supports the hypothesis that element size plays a significant role in successful transposition complex formation.

### Role of internal regions

**Fig. 2:**
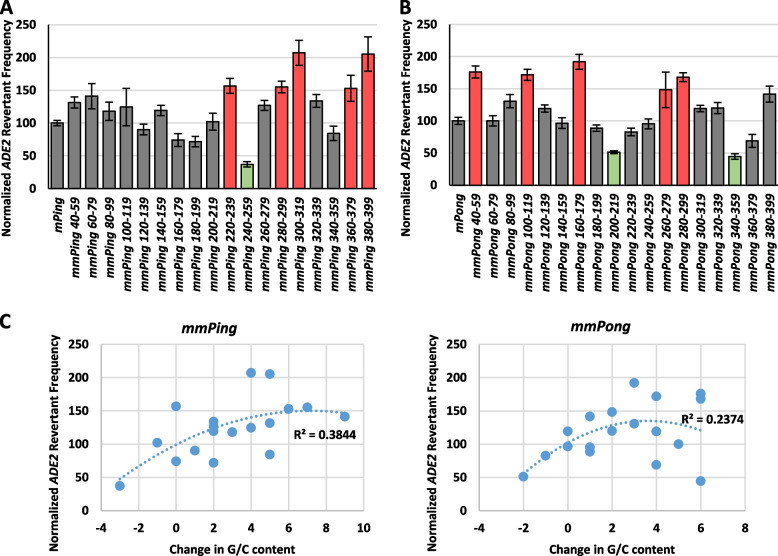
Normalized yeast transposition rates for systemic mutation of the internal sequences of *mPing* (**A**) and *mPong* (**B**). Columns represent the average and error bars represent standard error (*n* = 5-24). Columns in red represent elements with significantly increased transposition and those in green represent elements with significantly decreased transposition compared to the control. **C** The normalized transposition frequency of each mutant was graphed against the change in G/C content caused by the substitution of the wild type sequence with the systemic mutation sequence. Dotted line represents the line of best fit. R-squared values of the trendline are shown

A detailed study of a rice *Stowaway* MITE indicated that alteration of various internal sequences resulted in higher or lower transposition depending on where the sequence was changed [25]. We performed a similar analysis with the *mPing* and *mPong* elements to identify which regions function to regulate transposition. We systematically substituted 20 base increments from position 40 to 399 with a 20 bp sequence (CCCCTCTCTTAAGGTAGCCG, 60% GC rich) that contained an *IPpoI* restriction site to allow for confirmation by digestion. The transposition frequency of each mutated element was measured in the yeast transposition assay (Fig. 2). The majority of the mutant *mPing* (*mmPing)* and mutant *mPong* (*mmPong*) elements showed no change in transposition frequency, suggesting that these regions did not contain sequences required for transposition complex formation. However, the *mmPing 240-259*, *mmPong 200-219*, and *mmPong 340-359* elements showed significantly reduced transposition (*p* < 0.05) compared to the control elements (Fig. 2), suggesting that these regions innately play a role in transposition. In contrast, five *mmPing* elements and five *mmPong* elements showed significantly increased transposition (*p* < 0.05) compared to the control elements (Fig. 2), suggesting that the substitutions removed sequences that were innately inhibiting mobility.

The identified regulatory sequences’ positions appear to be unique for these two elements. For example, the *mPing* element has potential inhibitory regions clustered in the 3′ half of the element (Fig. 2A) while they are scattered more evenly throughout the *mPong* element (Fig. 2B). This finding that the location and sequences of these potential regulatory regions do not show any obvious correlation or homology indicated that these regulatory functions are achieved through general DNA features, such as flexibility or nucleosome association. To address this, we analyzed the GC content of each element compared to its relative transposition frequency (Fig. 2C). Overall, this analysis indicates that substitutions that increase the GC richness of the internal regions of the element resulted in higher transposition frequencies than substitutions that decrease GC richness. Increased GC content is known to be associated with increased nucleosome occupancy [28], suggesting that relatively small changes in the internal sequences of these MITES could increase histone binding, ultimately bringing the TIR sequences into closer proximity and affecting the efficiency of Transposase binding.

### Role of the terminal inverted repeats (TIRs)

**Fig. 3:**
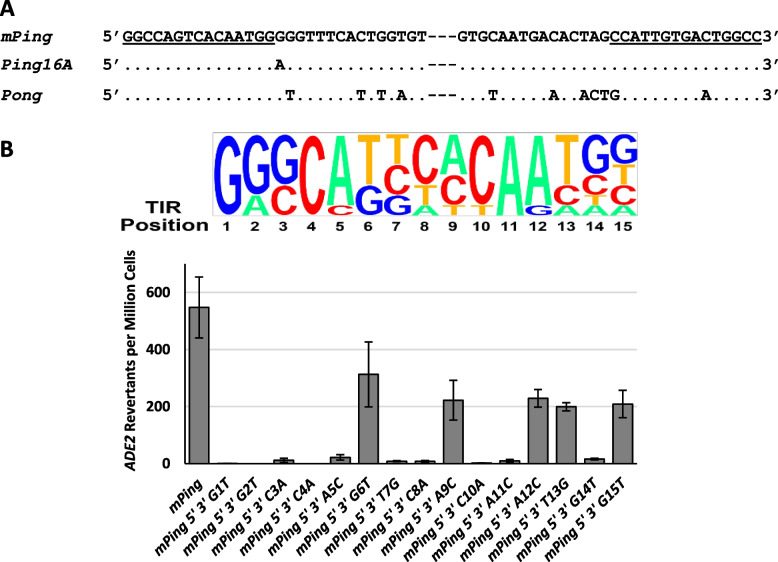
A sequence alignment (**A**) of the 30 bp at the 5′ and 3′ ends of the *mPing, Ping16A*, and *Pong* elements. Dots represent bases that are identical to *mPing*. Dashes represent the interior sequence. Underlined bases represent the TIRs. **B** Top panel- Pictogram representing the relative frequency of bases in the first 15 bases of the TIRs from selected *Tourist*-like MITEs (*mPing*, *Ditto 231*, *Youren 61*, *Stola 23*, *ID-4-X*, *Helia 1*, and *Tourist 1274*) from rice. Letter height indicates the frequency that that base is observed at that position (http://genes.mit.edu/pictogram.html). Bottom panel- Yeast transposition rates for *mPing* mutants with simultaneous mutations on both TIRs. Columns represent the average and the error bars represent standard error (*n* = 5-6)

Alignment of the 30 bp from each end of *mPing*, *Ping16A*, and *Pong* elements show that these related elements have very similar TIR sequences [defined as the terminal 15 bases, underlined] (Fig. 3A). The 15 bases on the 5′ end are entirely conserved amongst these elements. In comparison, the *Pong* 3′ TIR differs at positions 6 and 15 from the 3′ end. In addition, there are several variations in the sub-TIRs which may affect transposition. For example, previous studies pointed to the natural variation at the 16th base of the *Ping* element as key to its hypoactive transposition frequency [16]. A direct comparison of the *Ping* element with an A or a G at the 16th base confirmed that the 16A version had significantly less mobility (*p* < 0.05) in the yeast transposition assay (Supplemental Fig. 1). Thus, the *mPing* element, being derived from the higher activity version of *Ping* with a G at position 16, contributes to its burst in activity [16]. Based on this finding, we hypothesized that the other TIR variation between the *mPing* and *mPong* elements might play a role in regulating transposition activity.

Previous experiments focused on the role of the 5′ TIR showed that mutating each base (A to C, T to G, C to A, G to T) had a variety of effects, with some bases being highly required, while others were not [16]. We hypothesized that the importance of each base was related to its role in the interaction with the transposase proteins and predicted that important bases would be conserved. Figure 3B shows a pictogram of the TIR sequences of rice *Tourist*-like MITES similar to one created by Zhang, Jiang et al. 2004 [19], indicating both highly conserved positions (i.e., 1, 4, and 11) and relatively flexible positions (i.e., 14 and 15). We repeated the previously indicated experiments but simultaneously altering the TIR bases on both ends of the element (Fig. 3B). Mutation of both TIRs resulted in a more drastic reduction in transposition, with most mutations almost completely inhibiting activity and the remaining positions (6, 9, 12, 13, and 15) showing a significantly diminished frequency (*p* < 0.05) compared to the control (Fig. 3B). Some of the mutants that retained some activity can be explained by the fact that the base was mutated to one found in other Tourist MITES (i.e., G6T, A9C, G15T). Together, these results suggest that although some bases are more important than others, the entire TIR contributes to transposition complex formation and successful mobilization.

**Fig. 4:**
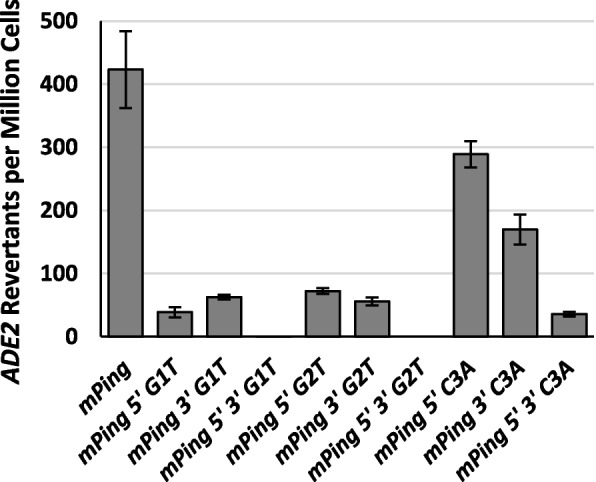
Yeast transposition rates for mutations in the 5′ TIR, 3′ TIR, or both TIRs at positions 1, 2, and 3 of *mPing*. Columns represent the average and error bars represent the standard error (*n* = 6)

To determine if one TIR is more critical for transposition than the other, we compared transposition rates for single base mutations on either the 3′ or 5′ TIR at positions 1, 2, and 3. This experiment showed that mutating either TIR produced similar effects (Fig. 4), suggesting that both ends play a similar role in transposition. Comparing the results for a single mutation to results for mutations at both TIRs indicates an additive effect resulting in a drastic decrease in the transposition rate when both TIRs are mutated (Fig. 4). This result is consistent with the reverse complimentary relationship of the two TIRs.

### Domain swapped elements

**Fig. 5:**
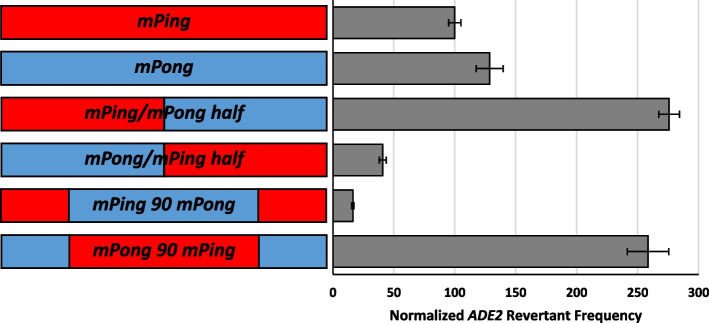
Normalized yeast transposition rates of *mPing* and *mPong* domain swapped elements. Element composition is shown on the left (red = *mPing*; blue = *mPong*). Columns represent the average and error bars represent the standard error (*n* = 6-12)

**Fig. 6:**
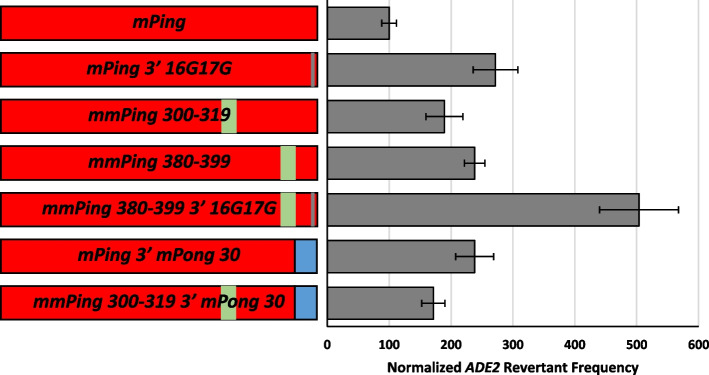
Normalized yeast transposition rates of *mPing* hyperactive mutants. Element composition is shown on the left (red = *mPing*; blue = *mPong*; green = 20 bp substitution; gray = 2 bp sub-TIR mutation). Columns represent the average and error bars represent the standard error (*n* = 6)

To further verify the role of the transposition regulating regions detected in *mPing* and *mPong*, we created four hybrid elements that contain segments of both *mPing* and *mPong* (Fig. 5)*.* Two of the domain-swapping constructs contained the first half (bases 1-215) of one element and the second half (bases 216-430) of the other element (*mPing/mPong half* and *mPong/mPing half*)*.* Two more domain-swapping constructs were made that contained 90 bases from both ends (bases 1-90 and 341-430) of one element and the central 250 bases (bases 91-340) of the other element (*mPing 90 mPong* and *mPong 90 mPing*). Transposition assays showed a dramatic increase in transposition of the *mPing/mPong half* compared to either parent element. In contrast, the converse structure *mPong/mPing half* showed a drastic decrease in transposition (Fig. 5). Similarly, we observed that the fusion construct *mPong 90 mPing,* which consists of the terminal 90 bases of *mPong* flanking the uninterrupted interior of *mPing,* showed hyperactive transposition, and the reverse combination was hypoactive (Fig. 5). These results suggest that the 3′ end of *mPing* is less efficient at forming functional transposition complexes than the 3′ end of *mPong*. This result was confirmed by testing the transposition of an element composed primarily of *mPing*, but with the 30 bases at the 5′ replaced by the corresponding *mPong* sequence. This element, called *mPing 3′ mPong 30*, showed higher transposition frequency than *mPing* (Fig. 6).

### Hyperactive elements

We hypothesized that altering the inefficient *mPing* 3′ end to mimic the efficient sequence found on the 5′ end could increase transposition frequency. Thus, we changed the bases at positions 16 and 17 on the 3′ sub-TIR to G (reading the reverse complement) to create the *mPing 3′ 16G17G* element. Yeast transposition assays showed that this element transposes at a significantly higher frequency (*p* < 0.05) than *mPing* (Fig. 6), indicating that the 16th and 17th bases are critical for regulating transposition on both ends of the element.

To determine if the interior sequences and TIRs have an additive effect on transposition, we made two elements with multiple transposition-promoting mutations. The *mmPing 380-399 3′ 16G17G* element has the internal substitution at 380-399 and the sub-TIR mutations at positions 16 and 17 in the 3′ end*.* The *mmPing 300-319 3′ mPong 30* element has the internal substitution at 300-319 and the last 30 bases from *mPong.* Our transposition assay shows that the *mmPing 380-399 3′ 16G17G* element with both mutations had significantly higher transposition rates (*p* < 0.05) than the elements with each mutation alone (Fig. 6). However, this additive was not observed for the *mmPing 300-319 3′ mPong 30* element, where combining the internal mutant and the TIR change did not significantly increase transposition compared to either mutation alone (Fig. 6). Together these results indicate that multiple factors contribute to active transposition complex formation, but additional experiments are needed to parse out the specific mechanisms.

## Discussion

The testing of full-length *Ping* and *Pong* elements in the yeast transposition assay (Fig. 1) allowed us to directly compare them to their corresponding MITEs. The finding that the larger elements exhibited lower transposition is not surprising, given that increased size has been shown to reduce transposition for other elements [29, 30]. However, this result supports the hypothesis that the observed difference in element copy number in rice is a direct consequence of transposition frequency. The observation that *Pong* transposes at a slightly higher frequency than *Ping16A* correlates with the difference in transposition observed for the *mPing* and *mPong* elements (Fig. 1). This suggests that our findings for these MITEs can be extrapolated to the larger elements. Overall, this also is consistent with the argument that much of the differences in transposition frequency between these elements are due to the internal and TIR sequences found in these MITEs.

Our systematic mutation of the *mPing* and *mPong* MITEs showed that they both contain multiple internal regions that significantly inhibit transposition (Fig. 2). The presence of these inhibiting regions supports a model in which transposable elements are selected for limited mobility, thus preventing excessive host damage and detection by host regulatory mechanisms. At the same time, the presence of important promoting regions in both elements indicates that the internal sequences of these elements also play a role in promoting transposition. One possibility is that these sequences recruit the ORF1 and TPase proteins or assist with transposition complex assembly. This type of internal binding is seen with the *Suppressor-mutator* element from maize, where the interaction between TnpA proteins and multiple binding sites internal to the TIRs is thought to bring the two TIRs together, facilitating transposition complex formation [31]. However, the fact that there is no specific transposition promoting internal sequence shared between the *mPing* and *mPong* MITEs suggests that these internal regions are not involved in sequence-specific roles. The correlation of transposition frequency with G/C content (Fig. 2C) suggests that the alteration of internal sequences may change the nucleosome occupancy. We hypothesize that increased nucleosome density at the TIRs may block ORF1 and TPase binding. On the other hand, internal nucleosomes may act to bring the TIRs together in closer proximity. Additional studies will be needed to clarify the role of nucleosome occupancy.

This study also provides additional insight into a previously identified mutant of *mPing,* named *mmPing20.* This hyperactive element contains seven internal base pair mutations [32]. Interestingly, five of the *mmPing20* mutations are within three transposition-repressing regions described in this study (Fig. 2A)*.* Of the other two base changes, one was in a transposition-promoting region, and the other was in a region that did not have a significant effect when mutated.

The role of the TIR sequences in forming the active transposition complex was also clarified by these experiments. First, by testing the same mutation in each TIR separately (Fig. 4), we demonstrated that both ends of the element play a similar role in transposition complex formation. In addition, these experiments showed that the effect of TIR alterations on both ends is additive. This finding is different from what is seen with some transposons, where one TIR appears to bind first in the process of transposition complex formation [i.e., *Sleeping Beauty* [33]]. It is unclear how this difference in transposition mechanism affects the success of these various elements. We hypothesize that since TIR sequences are important for transposition complex formation, TIR mutations will likely effect the mobility of most transposable elements.

By testing transposition rates in elements with simultaneous mutations on both ends, we exaggerated the effect of that base compared to previous results [16]. Thus, in Fig. 3, we see that all of *mPing’s* TIR bases contribute to the mobilization of the element. The most robust decreases in transposition were observed when highly conserved bases (i.e., positions 4 and 11) were changed, suggesting that the highly conserved bases play a critical role in the interaction with the ORF1 and TPase proteins in the transposition complex. The *Ping* ORF1 protein contains a GT1/Myb-like/SANt DNA binding domain that is likely to be involved in binding [19]. GT1/Myb-like/SANt DNA binding domains generally interact with about 6-8 bases [34, 35]. The fact that we see an effect over at least 15 bases suggests that the specificity-determining interaction may involve more than a single DNA binding domain. In addition, these results support the hypothesis that small changes in TIR sequences can manipulate element behavior, and they are likely selected for their ability to moderate transposition.

While both TIRs are essential for the formation of *mPing’s* transposition complex, mutation at one of the TIRs does not necessarily immobilize the element [16]. Based on the rice mutation rate, the TIRs should experience about 1.3 x 10-8 mutations per base per year [36]. These random mutations are likely the source of the differences observed in the *Pong* element, where the two TIRs do not match perfectly. Our data shows that TIR alterations may effectively immobilize the element, decrease the activity, or have no effect. In the case of the *mPong* element, “repairing” the *mPong* TIRs to be an exact inversion of each other did not affect transposition (unpublished). This result is consistent with the fact that there are multiple exact copies of the *Pong* element in the rice genome [19], suggesting relatively recent transposition.

Swapping portions from the related *mPing* and *mPong* elements allowed us to further evaluate the role of the sequence differences in transposition activity. While all the TIRs of these two elements were functional, we could determine that the 3′ end of the *mPing* element was not as efficient as the comparable TIR from *mPong* (Fig. 5). Combining the *mPong* 3′ TIR with the internal regions of *mPing* resulted in elements with significantly higher transposition than the parent elements (Fig. 6). This finding suggests that the *mPing* internal sequences are superior to *mPong* regarding the formation of an active transposition complex when paired with *Pong* TIRs. However, analysis of the internal 250 bp of these two elements shows no overall change in GC content, suggesting that the pattern or arrangement of bases may be important.

These results also provide additional insights into transposable element behavior as we look at the genomic context of these elements. We see that the *Ping* and *mPing* elements’ activity are moderated by having suboptimal TIRs. In contrast, the *Pong* element activity is potentially moderated by having suboptimal internal sequences. We previously showed that the ORF1 and TPase proteins are also not optimized for maximal transposition [14, 27]. Thus, our study of this group of elements has revealed multiple mechanisms by which these elements have been selected to self-regulate their activity. This effect does not appear to be exclusive to this class of elements, as evidence for moderately transposing elements has been seen for other classes of elements [i.e.*, Mariner* [37–39]]. However, as shown in these results, optimizing the sequences that prevent transposition results in hyperactivity. These hyperactive elements provide opportunities for developing efficient resources for plant gene discovery [32] and genome manipulation.

## Conclusions

Analysis of *mPing* and its related elements in the yeast transposition assay provides context to their overall transposition behavior in rice. Although host mechanisms play an important role in regulating *mPing* transposition [40], its sequence is likely selected to maintain moderate activity. Testing versions with modified sequences allowed us to pinpoint the regions that are important for transposition. These results indicate that both the TIRs, sub-TIRs, and internal regions play an important role in regulating the overall activity of *mPing, Ping,* and *Pong*. While the *mPing* sequence was sufficient to create a burst of transposition in rice, the creation of hyperactive versions indicates that it still encodes self-limiting sequences.

## Materials and methods

### Yeast strains

All experiments were done with the CB101 (Genotype: MATa ade2Δ::hphMX4 his3Δ1 leu2Δ0 met15Δ0 ura3Δ0 lys2Δ::ADE2*) or JIM17 (Genotype: *MATa ade2Δ::hphMX4 his3Δ1 leu2Δ0 met15Δ0 ura3Δ0*) yeast strains as described previously [41].

### Constructs

All constructs were made by ordering gBlocks™ (Integrated DNA Technologies, Inc.), amplifying the genes from genomic DNA from *Oryza sativa* cv. Nipponbare (*Ping16A* and *Pong*), or by high-fidelity PCR with primers containing the desired alterations. The fragments were co-transformed into yeast with *Hpa*I digested pWL89a as described previously [41, 42]. Plasmids were isolated by performing a yeast plasmid prep using a modified Zyppy Plasmid Miniprep (Zymo Research, Irvine, CA) protocol in which the yeast are lysed by vortexing with 425-600 μm glass beads for 3 minutes prior to adding neutralization buffer. The resulting plasmids are then transformed into *E. coli* to allow for plasmid amplification and sequencing. Element sequences are provided in Additional file 2.

### Yeast transposition assays

Transposition assays for Fig. 1 were performed in JIM17 using the pAG425 GAL Pong TPase L418A, L420A and pAG423 GAL ORF1 Shuffle1 NLS plasmids as previously described (Hancock, Zhang et al. 2010; Payero, Outten et al. 2016). All other transposition assays were performed in CB101 yeast containing the pAG425 GAL Pong TPase L418A, L420A and pAG413 GAL ORF1 Shuffle1 NLS plasmids as previously described [41]. All transposition assays were performed on 10 cm plates with 100 μl of culture plated on the galactose plates except for Fig. 6, in which 50 μl was plated from a 10^− 2^ dilution.

### Statistical analysis

All statistical analysis were performed using Graphpad Prism 9. Significance was calculated using a 1-way ANOVA with a Dunnett’s or Tukey’s multiple comparisons test or a 2-tailed Student’s *t* test. *P* less than 0.05 was considered statistically significant.

## Supplementary Information

**Additional file 1: Supplemental Fig. 1.** Yeast transposition rates of *Ping16A* and *Ping16G* elements. Columns represent the average and error bars represent the standard error (*n* = 6).

**Additional file 2.** FASTA file of the transposable elements tested in this study.

**Additional file 3.** Yeast excision frequency data sets.

## Data Availability

The dataset supporting the conclusions of this article are included within the article and its additional files. Plasmids used in the study are available through Addgene (ID 145794, 145795, 145787) or will be made available upon request.

## Abbreviations

TIR: Terminal Inverted Repeat
TPase: Transposase
MITE: Miniature Inverted Repeat Transposable Element
bp: Base pair

## References

[1] Feschotte C, Jiang N, Wessler SR (2002). Plant transposable elements: where genetics meets genomics. Nat Rev Genet.

[2] Oliver KR, McComb JA, Greene WK (2013). Transposable elements: powerful contributors to angiosperm evolution and diversity. Genome Biol Evol.

[3] Zhao DY, Ferguson AA, Jiang N (2016). What makes up plant genomes: the vanishing line between transposable elements and genes. Bba-Gene Regul Mech.

[4] Jiang N, Bao ZR, Zhang XY, Hirochika H, Eddy SR, McCouch SR (2003). An active DNA transposon family in rice. Nature..

[5] Naito K, Cho E, Yang GJ, Campbell MA, Yano K, Okumoto Y (2006). Dramatic amplification of a rice transposable element during recent domestication. Proc Natl Acad Sci U S A.

[6] Naito K, Zhang F, Tsukiyama T, Saito H, Hancock CN, Richardson AO (2009). Unexpected consequences of a sudden and massive transposon amplification on rice gene expression. Nature..

[7] Feschotte C, Zhang XY, Wessler SR, Craig NL, Craige R, Gellert M, Lambowitz A (2002). Miniature inverted-repeat transposable elements (MITEs) and their relationship with established DNA transposons. Mobile DNA II.

[8] Casa AM, Brouwer C, Nagel A, Wang LJ, Zhang Q, Kresovich S (2000). The MITE family *Heartbreaker* (*Hbr*): molecular markers in maize. Proc Natl Acad Sci U S A.

[9] Sampath P, Lee SC, Lee J, Izzah NK, Choi BS, Jin M (2013). Characterization of a new high copy *Stowaway* family MITE, *BRAMI-1* in Brassica genome. BMC Plant Biol.

[10] Yuan YW, Wessler SR (2011). The catalytic domain of all eukaryotic cut-and-paste transposase superfamilies. Proc Natl Acad Sci U S A.

[11] Rice PA, Baker TA (2001). Comparative architecture of transposase and integrase complexes. Nat Struct Biol.

[12] Ochmann MT, Ivics Z (2021). Jumping ahead with *sleeping beauty*: mechanistic insights into cut-and-paste transposition. Viruses-Basel..

[13] Sinzelle L, Kapitonov VV, Grzela DP, Jursch T, Jurka J, Izsvak Z (2008). Transposition of a reconstructed *Harbinger* element in human cells and functional homology with two transposon-derived cellular genes. Proc Natl Acad Sci U S A.

[14] Hancock CN, Zhang F, Wessler SR (2010). Transposition of the *tourist*-MITE *mPing* in yeast: an assay that retains key features of catalysis by the class 2 *PIF/Harbinger* superfamily. Mob DNA.

[15] Yang GJ, Zhang F, Hancock CN, Wessler SR (2007). Transposition of the rice miniature inverted repeat transposable element *mPing* in *Arabidopsis thaliana*. Proc Natl Acad Sci U S A.

[16] Chen J, Lu L, Benjamin J, Diaz S, Hancock CN, Stajich JE (2019). Tracking the origin of two genetic components associated with transposable element bursts in domesticated rice. Nat Commun.

[17] Lu L, Chen JF, Robb SMC, Okumoto Y, Stajich JE, Wessler SR (2017). Tracking the genome-wide outcomes of a transposable element burst over decades of amplification. Proc Natl Acad Sci U S A.

[18] Ngezahayo F, Xu CM, Wang HY, Jiang LL, Pang JS, Liu B (2009). Tissue culture-induced transpositional activity of *mPing* is correlated with cytosine methylation in rice. Bmc. Plant Biol.

[19] Zhang XY, Jiang N, Feschotte C, Wessler SR (2004). *PIF*- and *Pong*-like transposable elements: distribution, evolution and relationship with *Tourist-*like miniature inverted-repeat transposable elements. Genetics..

[20] Mullins MC, Rio DC, Rubin GM (1989). *cis*-acting DNA-sequence requirements for *P-element* transposition. Genes Dev.

[21] Cui ZB, Geurts AM, Liu GY, Kaufman CD, Hackett PB (2002). Structure-function analysis of the inverted terminal repeats of the *sleeping beauty* transposon. J Mol Biol.

[22] Fischer SEJ, van Luenen HGAM, Plasterk RHA (1999). Cis requirements for transposition of *Tc1*-like transposons in *C. elegans*. Mol Gen Genet.

[23] Huisman O, Errada PR, Signon L, Kleckner N (1989). Mutational analysis of *Is10*s outside end. EMBO J.

[24] Sakai J, Kleckner N (1996). Two classes of *Tn10* transposase mutants that suppress mutations in the *Tn10* terminal inverted repeat. Genetics..

[25] Yang GJ, Nagel DH, Feschotte C, Hancock CN, Wessler SR (2009). Tuned for transposition: molecular determinants underlying the hyperactivity of a *stowaway* MITE. Science..

[26] Li X, Harrell RA, Handler AM, Beam T, Hennessy K, Fraser MJ (2005). *piggyBac* internal sequences are necessary for efficient transformation of target genomes. Insect Mol Biol.

[27] Payero L, Outten G, Burckhalter C, Hancock CN (2016). Alteration of the *ping* and *pong* ORF1 proteins allows for hyperactive transposition of *mPing*. J South Carolina Acad Sci.

[28] Tillo D, Hughes TR (2009). G plus C content dominates intrinsic nucleosome occupancy. BMC Bioinformatics.

[29] Way JC, Kleckner N (1985). Transposition of plasmid-borne *Tn10* elements does not exhibit simple length-dependence. Genetics..

[30] Tosi LRO, Beverley SM (2000). Cis and trans factors affecting *Mos1 Mariner* evolution and transposition *in vitro*, and its potential for functional genomics. Nucleic Acids Res.

[31] Raina R, Schlappi M, Karunanandaa B, Elhofy A, Fedoroff N (1998). Concerted formation of macromolecular *suppressor-mutato*r transposition complexes. Proc Natl Acad Sci U S A.

[32] Johnson A, McAssey E, Diaz S, Reagin J, Redd PS, Parrilla DR (2021). Development of *mPing*-based activation tags for crop insertional mutagenesis. Plant Direct.

[33] Izsvak Z, Khare D, Behlke J, Heinemann U, Plasterk RH, Ivics Z (2002). Involvement of a bifunctional, paired-like DNA-binding domain and a transpositional enhancer in *sleeping beauty* transposition. J Biol Chem.

[34] Konig P, Fairall L, Rhodes D (1998). Sequence-specific DNA recognition by the Myb-like domain of the human telomere binding protein TRF1: a model for the protein-DNA complex. Nucleic Acids Res.

[35] Nagano Y, Inaba T, Furuhashi H, Sasaki Y (2001). Trihelix DNA-binding protein with specificities for two distinct cis-elements - both important for light down-regulated and dark-inducible gene expression in higher plants. J Biol Chem.

[36] Ma JX, Bennetzen JL (2004). Rapid recent growth and divergence of rice nuclear genomes. Proc Natl Acad Sci U S A.

[37] Zhou X, Xie J, Xu C, Cao X, Zou L-H, Zhou M (2022). Artificial optimization of bamboo *Ppmar2* transposase and host factors effects on *Ppmar2* transposition in yeast. Front Plant Sci.

[38] Mátés L, Chuah MK, Belay E, Jerchow B, Manoj N, Acosta-Sanchez A (2009). Molecular evolution of a novel hyperactive *sleeping beauty* transposase enables robust stable gene transfer in vertebrates. Nat Genet.

[39] Germon S, Bouchet N, Casteret S, Carpentier G, Adet J, Bigot Y (2009). *Mariner Mos1* transposase optimization by rational mutagenesis. Genetica..

[40] Tsukiyama T, Teramoto S, Yasuda K, Horibata A, Mori N, Okumoto Y (2013). Loss-of-function of a ubiquitin-related modifier promotes the mobilization of the active MITE *mPing*. Mol Plant.

[41] Gilbert DM, Bridges MC, Strother AE, Burckhalter CE, Burnette JM, Hancock CN (2015). Precise repair of *mPing* excision sites is facilitated by target site duplication derived microhomology. Mob DNA.

[42] Weil CF, Kunze R (2000). Transposition of maize *Ac*/*Ds* transposable elements in the yeast *Saccharomyces cerevisiae*. Nat Genet.

