# Supplementary figures and images for: Mobility of mPing and its associated elements is regulated by both internal and terminal sequences
Source: Mob DNA. 2023 Feb 11;14:1. doi: 10.1186/s13100-023-00289-3 (PMC9921582; doi:10.1186/s13100-023-00289-3)

## Slide 1
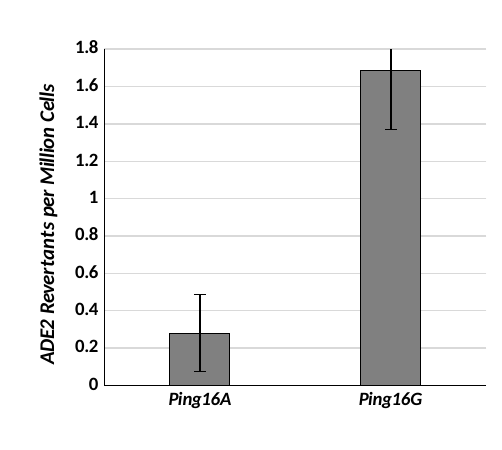

### Chart
| Category | |
|---|---|
| Ping16A | 0.2804834054834055 |
| Ping16G | 1.6846798388875996 |

Supplement: Supplementary file 1 — Additional file 1: Supplemental Fig. 1. Yeast transposition rates of Ping16A and Ping16G elements. Columns represent the average and error bars represent the standard error (n = 6). [file 13100_2023_289_MOESM1_ESM.pptx]
