# Supplementary material for: Mobility of mPing and its associated elements is regulated by both internal and terminal sequences
Source: Mob DNA. 2023 Feb 11;14:1. doi: 10.1186/s13100-023-00289-3 (PMC9921582; doi:10.1186/s13100-023-00289-3)
Supplement: Supplementary file 2 — Additional file 2. FASTA file of the transposable elements tested in this study. [file 13100_2023_289_MOESM2_ESM.docx]

>mmPing_40-59

GGCCAGTCACAATGGGGGTTTCACTGGTGTGTCATGCACCCCCTCTCTTAAGGTAGCCGAATAAAAAATGATTATTTGCATGAAATGGGGATGAGAGAGAAGGAAAGAGTTTCATCCTGGTGAAACTCGTCAGCGTCGTTTCCAAGTCCTCGGTAACAGAGTGAAACCCCCGTTGAGGCCGATTCGTTTCATTCACCGGATCTCTTGCGTCCGCCTCCGCCGTGCGACCTCTGCATTCTCCCGCGCCGCGCCGGATTTTGGGTACAAATGATCCCAGCAACTTGTATCAATTAAATGCTTTGCTTAGTCTTGGAAACGTCAAAGTGAAACCCCTCCACTGTGGGGATTGTTTCATAAAAGATTTCATTTGAGAGAAGATGGTATAATATTTTGGGTAGCCGTGCAATGACACTAGCCATTGTGACTGGCC

>mmPing_60-79

GGCCAGTCACAATGGGGGTTTCACTGGTGTGTCATGCACATTTAATAGGGGTAAGACTGCCCCTCTCTTAAGGTAGCCGATGAAATGGGGATGAGAGAGAAGGAAAGAGTTTCATCCTGGTGAAACTCGTCAGCGTCGTTTCCAAGTCCTCGGTAACAGAGTGAAACCCCCGTTGAGGCCGATTCGTTTCATTCACCGGATCTCTTGCGTCCGCCTCCGCCGTGCGACCTCTGCATTCTCCCGCGCCGCGCCGGATTTTGGGTACAAATGATCCCAGCAACTTGTATCAATTAAATGCTTTGCTTAGTCTTGGAAACGTCAAAGTGAAACCCCTCCACTGTGGGGATTGTTTCATAAAAGATTTCATTTGAGAGAAGATGGTATAATATTTTGGGTAGCCGTGCAATGACACTAGCCATTGTGACTGGCC

>mmPing_80-99

GGCCAGTCACAATGGGGGTTTCACTGGTGTGTCATGCACATTTAATAGGGGTAAGACTGAATAAAAAATGATTATTTGCCCCCTCTCTTAAGGTAGCCGAAGGAAAGAGTTTCATCCTGGTGAAACTCGTCAGCGTCGTTTCCAAGTCCTCGGTAACAGAGTGAAACCCCCGTTGAGGCCGATTCGTTTCATTCACCGGATCTCTTGCGTCCGCCTCCGCCGTGCGACCTCTGCATTCTCCCGCGCCGCGCCGGATTTTGGGTACAAATGATCCCAGCAACTTGTATCAATTAAATGCTTTGCTTAGTCTTGGAAACGTCAAAGTGAAACCCCTCCACTGTGGGGATTGTTTCATAAAAGATTTCATTTGAGAGAAGATGGTATAATATTTTGGGTAGCCGTGCAATGACACTAGCCATTGTGACTGGCC

>mmPing_100-119

GGCCAGTCACAATGGGGGTTTCACTGGTGTGTCATGCACATTTAATAGGGGTAAGACTGAATAAAAAATGATTATTTGCATGAAATGGGGATGAGAGAGCCCCTCTCTTAAGGTAGCCGGTGAAACTCGTCAGCGTCGTTTCCAAGTCCTCGGTAACAGAGTGAAACCCCCGTTGAGGCCGATTCGTTTCATTCACCGGATCTCTTGCGTCCGCCTCCGCCGTGCGACCTCTGCATTCTCCCGCGCCGCGCCGGATTTTGGGTACAAATGATCCCAGCAACTTGTATCAATTAAATGCTTTGCTTAGTCTTGGAAACGTCAAAGTGAAACCCCTCCACTGTGGGGATTGTTTCATAAAAGATTTCATTTGAGAGAAGATGGTATAATATTTTGGGTAGCCGTGCAATGACACTAGCCATTGTGACTGGCC

>mmPing_120-139

GGCCAGTCACAATGGGGGTTTCACTGGTGTGTCATGCACATTTAATAGGGGTAAGACTGAATAAAAAATGATTATTTGCATGAAATGGGGATGAGAGAGAAGGAAAGAGTTTCATCCTGCCCCTCTCTTAAGGTAGCCGTTCCAAGTCCTCGGTAACAGAGTGAAACCCCCGTTGAGGCCGATTCGTTTCATTCACCGGATCTCTTGCGTCCGCCTCCGCCGTGCGACCTCTGCATTCTCCCGCGCCGCGCCGGATTTTGGGTACAAATGATCCCAGCAACTTGTATCAATTAAATGCTTTGCTTAGTCTTGGAAACGTCAAAGTGAAACCCCTCCACTGTGGGGATTGTTTCATAAAAGATTTCATTTGAGAGAAGATGGTATAATATTTTGGGTAGCCGTGCAATGACACTAGCCATTGTGACTGGCC

>mmPing_140-159

GGCCAGTCACAATGGGGGTTTCACTGGTGTGTCATGCACATTTAATAGGGGTAAGACTGAATAAAAAATGATTATTTGCATGAAATGGGGATGAGAGAGAAGGAAAGAGTTTCATCCTGGTGAAACTCGTCAGCGTCGTCCCCTCTCTTAAGGTAGCCGAGTGAAACCCCCGTTGAGGCCGATTCGTTTCATTCACCGGATCTCTTGCGTCCGCCTCCGCCGTGCGACCTCTGCATTCTCCCGCGCCGCGCCGGATTTTGGGTACAAATGATCCCAGCAACTTGTATCAATTAAATGCTTTGCTTAGTCTTGGAAACGTCAAAGTGAAACCCCTCCACTGTGGGGATTGTTTCATAAAAGATTTCATTTGAGAGAAGATGGTATAATATTTTGGGTAGCCGTGCAATGACACTAGCCATTGTGACTGGCC

>mmPing_160-179

GGCCAGTCACAATGGGGGTTTCACTGGTGTGTCATGCACATTTAATAGGGGTAAGACTGAATAAAAAATGATTATTTGCATGAAATGGGGATGAGAGAGAAGGAAAGAGTTTCATCCTGGTGAAACTCGTCAGCGTCGTTTCCAAGTCCTCGGTAACAGCCCCTCTCTTAAGGTAGCCGCGATTCGTTTCATTCACCGGATCTCTTGCGTCCGCCTCCGCCGTGCGACCTCTGCATTCTCCCGCGCCGCGCCGGATTTTGGGTACAAATGATCCCAGCAACTTGTATCAATTAAATGCTTTGCTTAGTCTTGGAAACGTCAAAGTGAAACCCCTCCACTGTGGGGATTGTTTCATAAAAGATTTCATTTGAGAGAAGATGGTATAATATTTTGGGTAGCCGTGCAATGACACTAGCCATTGTGACTGGCC

>mmPing_180-199

GGCCAGTCACAATGGGGGTTTCACTGGTGTGTCATGCACATTTAATAGGGGTAAGACTGAATAAAAAATGATTATTTGCATGAAATGGGGATGAGAGAGAAGGAAAGAGTTTCATCCTGGTGAAACTCGTCAGCGTCGTTTCCAAGTCCTCGGTAACAGAGTGAAACCCCCGTTGAGGCCCCCTCTCTTAAGGTAGCCGATCTCTTGCGTCCGCCTCCGCCGTGCGACCTCTGCATTCTCCCGCGCCGCGCCGGATTTTGGGTACAAATGATCCCAGCAACTTGTATCAATTAAATGCTTTGCTTAGTCTTGGAAACGTCAAAGTGAAACCCCTCCACTGTGGGGATTGTTTCATAAAAGATTTCATTTGAGAGAAGATGGTATAATATTTTGGGTAGCCGTGCAATGACACTAGCCATTGTGACTGGCC

>mmPing_200-219

GGCCAGTCACAATGGGGGTTTCACTGGTGTGTCATGCACATTTAATAGGGGTAAGACTGAATAAAAAATGATTATTTGCATGAAATGGGGATGAGAGAGAAGGAAAGAGTTTCATCCTGGTGAAACTCGTCAGCGTCGTTTCCAAGTCCTCGGTAACAGAGTGAAACCCCCGTTGAGGCCGATTCGTTTCATTCACCGGCCCCTCTCTTAAGGTAGCCGCCGTGCGACCTCTGCATTCTCCCGCGCCGCGCCGGATTTTGGGTACAAATGATCCCAGCAACTTGTATCAATTAAATGCTTTGCTTAGTCTTGGAAACGTCAAAGTGAAACCCCTCCACTGTGGGGATTGTTTCATAAAAGATTTCATTTGAGAGAAGATGGTATAATATTTTGGGTAGCCGTGCAATGACACTAGCCATTGTGACTGGCC

>mmPing_220-239

GGCCAGTCACAATGGGGGTTTCACTGGTGTGTCATGCACATTTAATAGGGGTAAGACTGAATAAAAAATGATTATTTGCATGAAATGGGGATGAGAGAGAAGGAAAGAGTTTCATCCTGGTGAAACTCGTCAGCGTCGTTTCCAAGTCCTCGGTAACAGAGTGAAACCCCCGTTGAGGCCGATTCGTTTCATTCACCGGATCTCTTGCGTCCGCCTCCGCCCCTCTCTTAAGGTAGCCGCCCGCGCCGCGCCGGATTTTGGGTACAAATGATCCCAGCAACTTGTATCAATTAAATGCTTTGCTTAGTCTTGGAAACGTCAAAGTGAAACCCCTCCACTGTGGGGATTGTTTCATAAAAGATTTCATTTGAGAGAAGATGGTATAATATTTTGGGTAGCCGTGCAATGACACTAGCCATTGTGACTGGCC

>mmPing_240-259

GGCCAGTCACAATGGGGGTTTCACTGGTGTGTCATGCACATTTAATAGGGGTAAGACTGAATAAAAAATGATTATTTGCATGAAATGGGGATGAGAGAGAAGGAAAGAGTTTCATCCTGGTGAAACTCGTCAGCGTCGTTTCCAAGTCCTCGGTAACAGAGTGAAACCCCCGTTGAGGCCGATTCGTTTCATTCACCGGATCTCTTGCGTCCGCCTCCGCCGTGCGACCTCTGCATTCTCCCCTCTCTTAAGGTAGCCGGGGTACAAATGATCCCAGCAACTTGTATCAATTAAATGCTTTGCTTAGTCTTGGAAACGTCAAAGTGAAACCCCTCCACTGTGGGGATTGTTTCATAAAAGATTTCATTTGAGAGAAGATGGTATAATATTTTGGGTAGCCGTGCAATGACACTAGCCATTGTGACTGGCC

>mmPing_260-279

GGCCAGTCACAATGGGGGTTTCACTGGTGTGTCATGCACATTTAATAGGGGTAAGACTGAATAAAAAATGATTATTTGCATGAAATGGGGATGAGAGAGAAGGAAAGAGTTTCATCCTGGTGAAACTCGTCAGCGTCGTTTCCAAGTCCTCGGTAACAGAGTGAAACCCCCGTTGAGGCCGATTCGTTTCATTCACCGGATCTCTTGCGTCCGCCTCCGCCGTGCGACCTCTGCATTCTCCCGCGCCGCGCCGGATTTTCCCCTCTCTTAAGGTAGCCGACTTGTATCAATTAAATGCTTTGCTTAGTCTTGGAAACGTCAAAGTGAAACCCCTCCACTGTGGGGATTGTTTCATAAAAGATTTCATTTGAGAGAAGATGGTATAATATTTTGGGTAGCCGTGCAATGACACTAGCCATTGTGACTGGCC

>mmPing_280-299

GGCCAGTCACAATGGGGGTTTCACTGGTGTGTCATGCACATTTAATAGGGGTAAGACTGAATAAAAAATGATTATTTGCATGAAATGGGGATGAGAGAGAAGGAAAGAGTTTCATCCTGGTGAAACTCGTCAGCGTCGTTTCCAAGTCCTCGGTAACAGAGTGAAACCCCCGTTGAGGCCGATTCGTTTCATTCACCGGATCTCTTGCGTCCGCCTCCGCCGTGCGACCTCTGCATTCTCCCGCGCCGCGCCGGATTTTGGGTACAAATGATCCCAGCACCCCTCTCTTAAGGTAGCCGTTGCTTAGTCTTGGAAACGTCAAAGTGAAACCCCTCCACTGTGGGGATTGTTTCATAAAAGATTTCATTTGAGAGAAGATGGTATAATATTTTGGGTAGCCGTGCAATGACACTAGCCATTGTGACTGGCC

>mmPing_300-319

GGCCAGTCACAATGGGGGTTTCACTGGTGTGTCATGCACATTTAATAGGGGTAAGACTGAATAAAAAATGATTATTTGCATGAAATGGGGATGAGAGAGAAGGAAAGAGTTTCATCCTGGTGAAACTCGTCAGCGTCGTTTCCAAGTCCTCGGTAACAGAGTGAAACCCCCGTTGAGGCCGATTCGTTTCATTCACCGGATCTCTTGCGTCCGCCTCCGCCGTGCGACCTCTGCATTCTCCCGCGCCGCGCCGGATTTTGGGTACAAATGATCCCAGCAACTTGTATCAATTAAATGCTCCCCTCTCTTAAGGTAGCCGCAAAGTGAAACCCCTCCACTGTGGGGATTGTTTCATAAAAGATTTCATTTGAGAGAAGATGGTATAATATTTTGGGTAGCCGTGCAATGACACTAGCCATTGTGACTGGCC

>mmPing_300-319_3'_mPong_30

GGCCAGTCACAATGGGGGTTTCACTGGTGTGTCATGCACATTTAATAGGGGTAAGACTGAATAAAAAATGATTATTTGCATGAAATGGGGATGAGAGAGAAGGAAAGAGTTTCATCCTGGTGAAACTCGTCAGCGTCGTTTCCAAGTCCTCGGTAACAGAGTGAAACCCCCGTTGAGGCCGATTCGTTTCATTCACCGGATCTCTTGCGTCCGCCTCCGCCGTGCGACCTCTGCATTCTCCCGCGCCGCGCCGGATTTTGGGTACAAATGATCCCAGCAACTTGTATCAATTAAATGCTCCCCTCTCTTAAGGTAGCCGCAAAGTGAAACCCCTCCACTGTGGGGATTGTTTCATAAAAGATTTCATTTGAGAGAAGATGGTATAATATTTTGGGTAGCCGTGTAATGAAACACTGCATTGTGAATGGCC

>mmPing_320-339

GGCCAGTCACAATGGGGGTTTCACTGGTGTGTCATGCACATTTAATAGGGGTAAGACTGAATAAAAAATGATTATTTGCATGAAATGGGGATGAGAGAGAAGGAAAGAGTTTCATCCTGGTGAAACTCGTCAGCGTCGTTTCCAAGTCCTCGGTAACAGAGTGAAACCCCCGTTGAGGCCGATTCGTTTCATTCACCGGATCTCTTGCGTCCGCCTCCGCCGTGCGACCTCTGCATTCTCCCGCGCCGCGCCGGATTTTGGGTACAAATGATCCCAGCAACTTGTATCAATTAAATGCTTTGCTTAGTCTTGGAAACGTCCCCTCTCTTAAGGTAGCCGGTGGGGATTGTTTCATAAAAGATTTCATTTGAGAGAAGATGGTATAATATTTTGGGTAGCCGTGCAATGACACTAGCCATTGTGACTGGCC

>mmPing_340-359

GGCCAGTCACAATGGGGGTTTCACTGGTGTGTCATGCACATTTAATAGGGGTAAGACTGAATAAAAAATGATTATTTGCATGAAATGGGGATGAGAGAGAAGGAAAGAGTTTCATCCTGGTGAAACTCGTCAGCGTCGTTTCCAAGTCCTCGGTAACAGAGTGAAACCCCCGTTGAGGCCGATTCGTTTCATTCACCGGATCTCTTGCGTCCGCCTCCGCCGTGCGACCTCTGCATTCTCCCGCGCCGCGCCGGATTTTGGGTACAAATGATCCCAGCAACTTGTATCAATTAAATGCTTTGCTTAGTCTTGGAAACGTCAAAGTGAAACCCCTCCACTCCCCTCTCTTAAGGTAGCCGGATTTCATTTGAGAGAAGATGGTATAATATTTTGGGTAGCCGTGCAATGACACTAGCCATTGTGACTGGCC

>mmPing_360-379

GGCCAGTCACAATGGGGGTTTCACTGGTGTGTCATGCACATTTAATAGGGGTAAGACTGAATAAAAAATGATTATTTGCATGAAATGGGGATGAGAGAGAAGGAAAGAGTTTCATCCTGGTGAAACTCGTCAGCGTCGTTTCCAAGTCCTCGGTAACAGAGTGAAACCCCCGTTGAGGCCGATTCGTTTCATTCACCGGATCTCTTGCGTCCGCCTCCGCCGTGCGACCTCTGCATTCTCCCGCGCCGCGCCGGATTTTGGGTACAAATGATCCCAGCAACTTGTATCAATTAAATGCTTTGCTTAGTCTTGGAAACGTCAAAGTGAAACCCCTCCACTGTGGGGATTGTTTCATAAAACCCCTCTCTTAAGGTAGCCGGGTATAATATTTTGGGTAGCCGTGCAATGACACTAGCCATTGTGACTGGCC

>mmPing_380-399

GGCCAGTCACAATGGGGGTTTCACTGGTGTGTCATGCACATTTAATAGGGGTAAGACTGAATAAAAAATGATTATTTGCATGAAATGGGGATGAGAGAGAAGGAAAGAGTTTCATCCTGGTGAAACTCGTCAGCGTCGTTTCCAAGTCCTCGGTAACAGAGTGAAACCCCCGTTGAGGCCGATTCGTTTCATTCACCGGATCTCTTGCGTCCGCCTCCGCCGTGCGACCTCTGCATTCTCCCGCGCCGCGCCGGATTTTGGGTACAAATGATCCCAGCAACTTGTATCAATTAAATGCTTTGCATAGTCTTGGAAACGTCAAAGTGAAACCCCTCCACTGTGGGGATTGTTTCATAAAAGATTTCATTTGAGAGAAGATCCCCTCTCTTAAGGTAGCCGCGTGCAATGACACTAGCCATTGTGACTGGCC

>mmPing_380-399_3'_16G17G

GGCCAGTCACAATGGGGGTTTCACTGGTGTGTCATGCACATTTAATAGGGGTAAGACTGAATAAAAAATGATTATTTGCATGAAATGGGGATGAGAGAGAAGGAAAGAGTTTCATCCTGGTGAAACTCGTCAGCGTCGTTTCCAAGTCCTCGGTAACAGAGTGAAACCCCCGTTGAGGCCGATTCGTTTCATTCACCGGATCTCTTGCGTCCGCCTCCGCCGTGCGACCTCTGCATTCTCCCGCGCCGCGCCGGATTTTGGGTACAAATGATCCCAGCAACTTGTATCAATTAAATGCTTTGCATAGTCTTGGAAACGTCAAAGTGAAACCCCTCCACTGTGGGGATTGTTTCATAAAAGATTTCATTTGAGAGAAGATCCCCTCTCTTAAGGTAGCCGCGTGCAATGACACTCCCCATTGTGACTGGCC

>mmPong_40-59

GGCCAGTCACAATGGGTGTTTCATTTGAGTGTCATGCGCCCCCTCTCTTAAGGTAGCCGAGCAAAAGAGCAATATTTGCATGAAATGGGTAGGAGAGAGAGTAAACTCGTTTCACCATGGTGACACGAGATAGCGCCGTTTCCCAGGTCGCTGAAACGGGGTGAAACAGCATTGAGAGTTCATCGTTTCACCTCCGGGATCCCGTGCGAGCGCTGCTCTTCGCCATCTTCGCGCGCATCGCCGGATTCTTCCACATACGATCCCACTATGTGGCTGGAATTAAATGCCTTGAATTTGCATTGGAAACGCTAGAGTGAAACACAGCATTGAGAAGGTCTGTTTCATTGTACGTTTCAACTTGTTTCATCTTCGTTTCAGCTGATGTGGCGTCTGGGAAACAGTGTAATGAAACACTGCATTGTGAATGGCC

>mmPong_60-79

GGCCAGTCACAATGGGTGTTTCATTTGAGTGTCATGCGCATTTAATACAGTGACAAGTCCCCCTCTCTTAAGGTAGCCGATGAAATGGGTAGGAGAGAGAGTAAACTCGTTTCACCATGGTGACACGAGATAGCGCCGTTTCCCAGGTCGCTGAAACGGGGTGAAACAGCATTGAGAGTTCATCGTTTCACCTCCGGGATCCCGTGCGAGCGCTGCTCTTCGCCATCTTCGCGCGCATCGCCGGATTCTTCCACATACGATCCCACTATGTGGCTGGAATTAAATGCCTTGAATTTGCATTGGAAACGCTAGAGTGAAACACAGCATTGAGAAGGTCTGTTTCATTGTACGTTTCAACTTGTTTCATCTTCGTTTCAGCTGATGTGGCGTCTGGGAAACAGTGTAATGAAACACTGCATTGTGAATGGCC

>mmPong_80-99

GGCCAGTCACAATGGGTGTTTCATTTGAGTGTCATGCGCATTTAATACAGTGACAAGTCAGCAAAAGAGCAATATTTGCCCCCTCTCTTAAGGTAGCCGAGTAAACTCGTTTCACCATGGTGACACGAGATAGCGCCGTTTCCCAGGTCGCTGAAACGGGGTGAAACAGCATTGAGAGTTCATCGTTTCACCTCCGGGATCCCGTGCGAGCGCTGCTCTTCGCCATCTTCGCGCGCATCGCCGGATTCTTCCACATACGATCCCACTATGTGGCTGGAATTAAATGCCTTGAATTTGCATTGGAAACGCTAGAGTGAAACACAGCATTGAGAAGGTCTGTTTCATTGTACGTTTCAACTTGTTTCATCTTCGTTTCAGCTGATGTGGCGTCTGGGAAACAGTGTAATGAAACACTGCATTGTGAATGGCC

>mmPong_100-119

GGCCAGTCACAATGGGTGTTTCATTTGAGTGTCATGCGCATTTAATACAGTGACAAGTCAGCAAAAGAGCAATATTTGCATGAAATGGGTAGGAGAGAGCCCCTCTCTTAAGGTAGCCGGTGACACGAGATAGCGCCGTTTCCCAGGTCGCTGAAACGGGGTGAAACAGCATTGAGAGTTCATCGTTTCACCTCCGGGATCCCGTGCGAGCGCTGCTCTTCGCCATCTTCGCGCGCATCGCCGGATTCTTCCACATACGATCCCACTATGTGGCTGGAATTAAATGCCTTGAATTTGCATTGGAAACGCTAGAGTGAAACACAGCATTGAGAAGGTCTGTTTCATTGTACGTTTCAACTTGTTTCATCTTCGTTTCAGCTGATGTGGCGTCTGGGAAACAGTGTAATGAAACACTGCATTGTGAATGGCC

>mmPong_120-139

GGCCAGTCACAATGGGTGTTTCATTTGAGTGTCATGCGCATTTAATACAGTGACAAGTCAGCAAAAGAGCAATATTTGCATGAAATGGGTAGGAGAGAGAGTAAACTCGTTTCACCATGCCCCTCTCTTAAGGTAGCCGTTCCCAGGTCGCTGAAACGGGGTGAAACAGCATTGAGAGTTCATCGTTTCACCTCCGGGATCCCGTGCGAGCGCTGCTCTTCGCCATCTTCGCGCGCATCGCCGGATTCTTCCACATACGATCCCACTATGTGGCTGGAATTAAATGCCTTGAATTTGCATTGGAAACGCTAGAGTGAAACACAGCATTGAGAAGGTCTGTTTCATTGTACGTTTCAACTTGTTTCATCTTCGTTTCAGCTGATGTGGCGTCTGGGAAACAGTGTAATGAAACACTGCATTGTGAATGGCC

>mmPong_140-159

GGCCAGTCACAATGGGTGTTTCATTTGAGTGTCATGCGCATTTAATACAGTGACAAGTCAGCAAAAGAGCAATATTTGCATGAAATGGGTAGGAGAGAGAGTAAACTCGTTTCACCATGGTGACACGAGATAGCGCCGTCCCCTCTCTTAAGGTAGCCGGGTGAAACAGCATTGAGAGTTCATCGTTTCACCTCCGGGATCCCGTGCGAGCGCTGCTCTTCGCCATCTTCGCGCGCATCGCCGGATTCTTCCACATACGATCCCACTATGTGGCTGGAATTAAATGCCTTGAATTTGCATTGGAAACGCTAGAGTGAAACACAGCATTGAGAAGGTCTGTTTCATTGTACGTTTCAACTTGTTTCATCTTCGTTTCAGCTGATGTGGCGTCTGGGAAACAGTGTAATGAAACACTGCATTGTGAATGGCC

>mmPong_160-179

GGCCAGTCACAATGGGTGTTTCATTTGAGTGTCATGCGCATTTAATACAGTGACAAGTCAGCAAAAGAGCAATATTTGCATGAAATGGGTAGGAGAGAGAGTAAACTCGTTTCACCATGGTGACACGAGATAGCGCCGTTTCCCAGGTCGCTGAAACGGCCCCTCTCTTAAGGTAGCCGTCATCGTTTCACCTCCGGGATCCCGTGCGAGCGCTGCTCTTCGCCATCTTCGCGCGCATCGCCGGATTCTTCCACATACGATCCCACTATGTGGCTGGAATTAAATGCCTTGAATTTGCATTGGAAACGCTAGAGTGAAACACAGCATTGAGAAGGTCTGTTTCATTGTACGTTTCAACTTGTTTCATCTTCGTTTCAGCTGATGTGGCGTCTGGGAAACAGTGTAATGAAACACTGCATTGTGAATGGCC

>mmPong_180-199

GGCCAGTCACAATGGGTGTTTCATTTGAGTGTCATGCGCATTTAATACAGTGACAAGTCAGCAAAAGAGCAATATTTGCATGAAATGGGTAGGAGAGAGAGTAAACTCGTTTCACCATGGTGACACGAGATAGCGCCGTTTCCCAGGTCGCTGAAACGGGGTGAAACAGCATTGAGAGTCCCCTCTCTTAAGGTAGCCGTCCCGTGCGAGCGCTGCTCTTCGCCATCTTCGCGCGCATCGCCGGATTCTTCCACATACGATCCCACTATGTGGCTGGAATTAAATGCCTTGAATTTGCATTGGAAACGCTAGAGTGAAACACAGCATTGAGAAGGTCTGTTTCATTGTACGTTTCAACTTGTTTCATCTTCGTTTCAGCTGATGTGGCGTCTGGGAAACAGTGTAATGAAACACTGCATTGTGAATGGCC

>mmPong_200-219

GGCCAGTCACAATGGGTGTTTCATTTGAGTGTCATGCGCATTTAATACAGTGACAAGTCAGCAAAAGAGCAATATTTGCATGAAATGGGTAGGAGAGAGAGTAAACTCGTTTCACCATGGTGACACGAGATAGCGCCGTTTCCCAGGTCGCTGAAACGGGGTGAAACAGCATTGAGAGTTCATCGTTTCACCTCCGGGACCCCTCTCTTAAGGTAGCCGTCGCCATCTTCGCGCGCATCGCCGGATTCTTCCACATACGATCCCACTATGTGGCTGGAATTAAATGCCTTGAATTTGCATTGGAAACGCTAGAGTGAAACACAGCATTGAGAAGGTCTGTTTCATTGTACGTTTCAACTTGTTTCATCTTCGTTTCAGCTGATGTGGCGTCTGGGAAACAGTGTAATGAAACACTGCATTGTGAATGGCC

>mmPong_220-239

GGCCAGTCACAATGGGTGTTTCATTTGAGTGTCATGCGCATTTAATACAGTGACAAGTCAGCAAAAGAGCAATATTTGCATGAAATGGGTAGGAGAGAGAGTAAACTCGTTTCACCATGGTGACACGAGATAGCGCCGTTTCCCAGGTCGCTGAAACGGGGTGAAACAGCATTGAGAGTTCATCGTTTCACCTCCGGGATCCCGTGCGAGCGCTGCTCTCCCCTCTCTTAAGGTAGCCGGCCGGATTCTTCCACATACGATCCCACTATGTGGCTGGAATTAAATGCCTTGAATTTGCATTGGAAACGCTAGAGTGAAACACAGCATTGAGAAGGTCTGTTTCATTGTACGTTTCAACTTGTTTCATCTTCGTTTCAGCTGATGTGGCGTCTGGGAAACAGTGTAATGAAACACTGCATTGTGAATGGCC

>mmPong_240-259

GGCCAGTCACAATGGGTGTTTCATTTGAGTGTCATGCGCATTTAATACAGTGACAAGTCAGCAAAAGAGCAATATTTGCATGAAATGGGTAGGAGAGAGAGTAAACTCGTTTCACCATGGTGACACGAGATAGCGCCGTTTCCCAGGTCGCTGAAACGGGGTGAAACAGCATTGAGAGTTCATCGTTTCACCTCCGGGATCCCGTGCGAGCGCTGCTCTTCGCCATCTTCGCGCGCATCCCCCTCTCTTAAGGTAGCCGATCCCACTATGTGGCTGGAATTAAATGCCTTGAATTTGCATTGGAAACGCTAGAGTGAAACACAGCATTGAGAAGGTCTGTTTCATTGTACGTTTCAACTTGTTTCATCTTCGTTTCAGCTGATGTGGCGTCTGGGAAACAGTGTAATGAAACACTGCATTGTGAATGGCC

>mmPong_260-279

GGCCAGTCACAATGGGTGTTTCATTTGAGTGTCATGCGCATTTAATACAGTGACAAGTCAGCAAAAGAGCAATATTTGCATGAAATGGGTAGGAGAGAGAGTAAACTCGTTTCACCATGGTGACACGAGATAGCGCCGTTTCCCAGGTCGCTGAAACGGGGTGAAACAGCATTGAGAGTTCATCGTTTCACCTCCGGGATCCCGTGCGAGCGCTGCTCTTCGCCATCTTCGCGCGCATCGCCGGATTCTTCCACATACGCCCCTCTCTTAAGGTAGCCGTTAAATGCCTTGAATTTGCATTGGAAACGCTAGAGTGAAACACAGCATTGAGAAGGTCTGTTTCATTGTACGTTTCAACTTGTTTCATCTTCGTTTCAGCTGATGTGGCGTCTGGGAAACAGTGTAATGAAACACTGCATTGTGAATGGCC

>mmPong_280-299

GGCCAGTCACAATGGGTGTTTCATTTGAGTGTCATGCGCATTTAATACAGTGACAAGTCAGCAAAAGAGCAATATTTGCATGAAATGGGTAGGAGAGAGAGTAAACTCGTTTCACCATGGTGACACGAGATAGCGCCGTTTCCCAGGTCGCTGAAACGGGGTGAAACAGCATTGAGAGTTCATCGTTTCACCTCCGGGATCCCGTGCGAGCGCTGCTCTTCGCCATCTTCGCGCGCATCGCCGGATTCTTCCACATACGATCCCACTATGTGGCTGGAACCCCTCTCTTAAGGTAGCCGTTGGAAACGCTAGAGTGAAACACAGCATTGAGAAGGTCTGTTTCATTGTACGTTTCAACTTGTTTCATCTTCGTTTCAGCTGATGTGGCGTCTGGGAAACAGTGTAATGAAACACTGCATTGTGAATGGCC

>mmPong_300-319

GGCCAGTCACAATGGGTGTTTCATTTGAGTGTCATGCGCATTTAATACAGTGACAAGTCAGCAAAAGAGCAATATTTGCATGAAATGGGTAGGAGAGAGAGTAAACTCGTTTCACCATGGTGACACGAGATAGCGCCGTTTCCCAGGTCGCTGAAACGGGGTGAAACAGCATTGAGAGTTCATCGTTTCACCTCCGGGATCCCGTGCGAGCGCTGCTCTTCGCCATCTTCGCGCGCATCGCCGGATTCTTCCACATACGATCCCACTATGTGGCTGGAATTAAATGCCTTGAATTTGCACCCCTCTCTTAAGGTAGCCGCACAGCATTGAGAAGGTCTGTTTCATTGTACGTTTCAACTTGTTTCATCTTCGTTTCAGCTGATGTGGCGTCTGGGAAACAGTGTAATGAAACACTGCATTGTGAATGGCC

>mmPong_320-339

GGCCAGTCACAATGGGTGTTTCATTTGAGTGTCATGCGCATTTAATACAGTGACAAGTCAGCAAAAGAGCAATATTTGCATGAAATGGGTAGGAGAGAGAGTAAACTCGTTTCACCATGGTGACACGAGATAGCGCCGTTTCCCAGGTCGCTGAAACGGGGTGAAACAGCATTGAGAGTTCATCGTTTCACCTCCGGGATCCCGTGCGAGCGCTGCTCTTCGCCATCTTCGCGCGCATCGCCGGATTCTTCCACATACGATCCCACTATGTGGCTGGAATTAAATGCCTTGAATTTGCATTGGAAACGCTAGAGTGAAACCCCTCTCTTAAGGTAGCCGTTTCATTGTACGTTTCAACTTGTTTCATCTTCGTTTCAGCTGATGTGGCGTCTGGGAAACAGTGTAATGAAACACTGCATTGTGAATGGCC

>mmPong_340-359

GGCCAGTCACAATGGGTGTTTCATTTGAGTGTCATGCGCATTTAATACAGTGACAAGTCAGCAAAAGAGCAATATTTGCATGAAATGGGTAGGAGAGAGAGTAAACTCGTTTCACCATGGTGACACGAGATAGCGCCGTTTCCCAGGTCGCTGAAACGGGGTGAAACAGCATTGAGAGTTCATCGTTTCACCTCCGGGATCCCGTGCGAGCGCTGCTCTTCGCCATCTTCGCGCGCATCGCCGGATTCTTCCACATACGATCCCACTATGTGGCTGGAATTAAATGCCTTGAATTTGCATTGGAAACGCTAGAGTGAAACACAGCATTGAGAAGGTCTGCCCCTCTCTTAAGGTAGCCGTGTTTCATCTTCGTTTCAGCTGATGTGGCGTCTGGGAAACAGTGTAATGAAACACTGCATTGTGAATGGCC

>mmPong_360-379

GGCCAGTCACAATGGGTGTTTCATTTGAGTGTCATGCGCATTTAATACAGTGACAAGTCAGCAAAAGAGCAATATTTGCATGAAATGGGTAGGAGAGAGAGTAAACTCGTTTCACCATGGTGACACGAGATAGCGCCGTTTCCCAGGTCGCTGAAACGGGGTGAAACAGCATTGAGAGTTCATCGTTTCACCTCCGGGATCCCGTGCGAGCGCTGCTCTTCGCCATCTTCGCGCGCATCGCCGGATTCTTCCACATACGATCCCACTATGTGGCTGGAATTAAATGCCTTGAATTTGCATTGGAAACGCTAGAGTGAAACACAGCATTGAGAAGGTCTGTTTCATTGTACGTTTCAACTCCCCTCTCTTAAGGTAGCCGTGATGTGGCGTCTGGGAAACAGTGTAATGAAACACTGCATTGTGAATGGCC

>mmPong_380-399

GGCCAGTCACAATGGGTGTTTCATTTGAGTGTCATGCGCATTTAATACAGTGACAAGTCAGCAAAAGAGCAATATTTGCATGAAATGGGTAGGAGAGAGAGTAAACTCGTTTCACCATGGTGACACGAGATAGCGCCGTTTCCCAGGTCGCTGAAACGGGGTGAAACAGCATTGAGAGTTCATCGTTTCACCTCCGGGATCCCGTGCGAGCGCTGCTCTTCGCCATCTTCGCGCGCATCGCCGGATTCTTCCACATACGATCCCACTATGTGGCTGGAATTAAATGCCTTGAATTTGCATTGGAAACGCTAGAGTGAAACACAGCATTGAGAAGGTCTGTTTCATTGTACGTTTCAACTTGTTTCATCTTCGTTTCAGCCCCCTCTCTTAAGGTAGCCGAGTGTAATGAAACACTGCATTGTGAATGGCC

>mPing

GGCCAGTCACAATGGGGGTTTCACTGGTGTGTCATGCACATTTAATAGGGGTAAGACTGAATAAAAAATGATTATTTGCATGAAATGGGGATGAGAGAGAAGGAAAGAGTTTCATCCTGGTGAAACTCGTCAGCGTCGTTTCCAAGTCCTCGGTAACAGAGTGAAACCCCCGTTGAGGCCGATTCGTTTCATTCACCGGATCTCTTGCGTCCGCCTCCGCCGTGCGACCTCTGCATTCTCCCGCGCCGCGCCGGATTTTGGGTACAAATGATCCCAGCAACTTGTATCAATTAAATGCTTTGCTTAGTCTTGGAAACGTCAAAGTGAAACCCCTCCACTGTGGGGATTGTTTCATAAAAGATTTCATTTGAGAGAAGATGGTATAATATTTTGGGTAGCCGTGCAATGACACTAGCCATTGTGACTGGCC

>mPing_3'_16G17G

GGCCAGTCACAATGGGGGTTTCACTGGTGTGTCATGCACATTTAATAGGGGTAAGACTGAATAAAAAATGATTATTTGCATGAAATGGGGATGAGAGAGAAGGAAAGAGTTTCATCCTGGTGAAACTCGTCAGCGTCGTTTCCAAGTCCTCGGTAACAGAGTGAAACCCCCGTTGAGGCCGATTCGTTTCATTCACCGGATCTCTTGCGTCCGCCTCCGCCGTGCGACCTCTGCATTCTCCCGCGCCGCGCCGGATTTTGGGTACAAATGATCCCAGCAACTTGTATCAATTAAATGCTTTGCATAGTCTTGGAAACGTCAAAGTGAAACCCCTCCACTGTGGGGATTGTTTCATAAAAGATTTCATTTGAGAGAAGATGGTATAATATTTTGGGTAGCCGTGCAATGACACTCCCCATTGTGACTGGCC

>mPing_3'_C3A

GGCCAGTCACAATGGGGGTTTCACTGGTGTGTCATGCACATTTAATAGGGGTAAGACTGAATAAAAAATGATTATTTGCATGAAATGGGGATGAGAGAGAAGGAAAGAGTTTCATCCTGGTGAAACTCGTCAGCGTCGTTTCCAAGTCCTCGGTAACAGAGTGAAACCCCCGTTGAGGCCGATTCGTTTCATTCACCGGATCTCTTGCGTCCGCCTCCGCCGTGCGACCTCTGCATTCTCCCGCGCCGCGCCGGATTTTGGGTACAAATGATCCCAGCAACTTGTATCAATTAAATGCTTTGCTTAGTCTTGGAAACGTCAAAGTGAAACCCCTCCACTGTGGGGATTGTTTCATAAAAGATTTCATTTGAGAGAAGATGGTATAATATTTTGGGTAGCCGTGCAATGACACTAGCCATTGTGACTGTCC

>mPing_3'_G1T

GGCCAGTCACAATGGGGGTTTCACTGGTGTGTCATGCACATTTAATAGGGGTAAGACTGAATAAAAAATGATTATTTGCATGAAATGGGGATGAGAGAGAAGGAAAGAGTTTCATCCTGGTGAAACTCGTCAGCGTCGTTTCCAAGTCCTCGGTAACAGAGTGAAACCCCCGTTGAGGCCGATTCGTTTCATTCACCGGATCTCTTGCGTCCGCCTCCGCCGTGCGACCTCTGCATTCTCCCGCGCCGCGCCGGATTTTGGGTACAAATGATCCCAGCAACTTGTATCAATTAAATGCTTTGCTTAGTCTTGGAAACGTCAAAGTGAAACCCCTCCACTGTGGGGATTGTTTCATAAAAGATTTCATTTGAGAGAAGATGGTATAATATTTTGGGTAGCCGTGCAATGACACTAGCCATTGTGACTGGCA

>mPing_3'_G2T

GGCCAGTCACAATGGGGGTTTCACTGGTGTGTCATGCACATTTAATAGGGGTAAGACTGAATAAAAAATGATTATTTGCATGAAATGGGGATGAGAGAGAAGGAAAGAGTTTCATCCTGGTGAAACTCGTCAGCGTCGTTTCCAAGTCCTCGGTAACAGAGTGAAACCCCCGTTGAGGCCGATTCGTTTCATTCACCGGATCTCTTGCGTCCGCCTCCGCCGTGCGACCTCTGCATTCTCCCGCGCCGCGCCGGATTTTGGGTACAAATGATCCCAGCAACTTGTATCAATTAAATGCTTTGCTTAGTCTTGGAAACGTCAAAGTGAAACCCCTCCACTGTGGGGATTGTTTCATAAAAGATTTCATTTGAGAGAAGATGGTATAATATTTTGGGTAGCCGTGCAATGACACTAGCCATTGTGACTGGAC

>mPing_3'_mPong_30

GGCCAGTCACAATGGGGGTTTCACTGGTGTGTCATGCACATTTAATAGGGGTAAGACTGAATAAAAAATGATTATTTGCATGAAATGGGGATGAGAGAGAAGGAAAGAGTTTCATCCTGGTGAAACTCGTCAGCGTCGTTTCCAAGTCCTCGGTAACAGAGTGAAACCCCCGTTGAGGCCGATTCGTTTCATTCACCGGATCTCTTGCGTCCGCCTCCGCCGTGCGACCTCTGCATTCTCCCGCGCCGCGCCGGATTTTGGGTACAAATGATCCCAGCAACTTGTATCAATTAAATGCTTTGCTTAGTCTTGGAAACGTCAAAGTGAAACCCCTCCACTGTGGGGATTGTTTCATAAAAGATTTCATTTGAGAGAAGATGGTATAATATTTTGGGTAGCCGTGTAATGAAACACTGCATTGTGAATGGCC

>mPing_5'_3'_A5C

GGCCCGTCACAATGGGGGTTTCACTGGTGTGTCATGCACATTTAATAGGGGTAAGACTGAATAAAAAATGATTATTTGCATGAAATGGGGATGAGAGAGAAGGAAAGAGTTTCATCCTGGTGAAACTCGTCAGCGTCGTTTCCAAGTCCTCGGTAACAGAGTGAAACCCCCGTTGAGGCCGATTCGTTTCATTCACCGGATCTCTTGCGTCCGCCTCCGCCGTGCGACCTCTGCATTCTCCCGCGCCGCGCCGGATTTTGGGTACAAATGATCCCAGCAACTTGTATCAATTAAATGCTTTGCTTAGTCTTGGAAACGTCAAAGTGAAACCCCTCCACTGTGGGGATTGTTTCATAAAAGATTTCATTTGAGAGAAGATGGTATAATATTTTGGGTAGCCGTGCAATGACACTAGCCATTGTGACGGGCC

>mPing_5'_3'_A9C

GGCCAGTCCCAATGGGGGTTTCACTGGTGTGTCATGCACATTTAATAGGGGTAAGACTGAATAAAAAATGATTATTTGCATGAAATGGGGATGAGAGAGAAGGAAAGAGTTTCATCCTGGTGAAACTCGTCAGCGTCGTTTCCAAGTCCTCGGTAACAGAGTGAAACCCCCGTTGAGGCCGATTCGTTTCATTCACCGGATCTCTTGCGTCCGCCTCCGCCGTGCGACCTCTGCATTCTCCCGCGCCGCGCCGGATTTTGGGTACAAATGATCCCAGCAACTTGTATCAATTAAATGCTTTGCTTAGTCTTGGAAACGTCAAAGTGAAACCCCTCCACTGTGGGGATTGTTTCATAAAAGATTTCATTTGAGAGAAGATGGTATAATATTTTGGGTAGCCGTGCAATGACACTAGCCATTGGGACTGGCC

>mPing_5'_3'_A11C

GGCCAGTCACCATGGGGGTTTCACTGGTGTGTCATGCACATTTAATAGGGGTAAGACTGAATAAAAAATGATTATTTGCATGAAATGGGGATGAGAGAGAAGGAAAGAGTTTCATCCTGGTGAAACTCGTCAGCGTCGTTTCCAAGTCCTCGGTAACAGAGTGAAACCCCCGTTGAGGCCGATTCGTTTCATTCACCGGATCTCTTGCGTCCGCCTCCGCCGTGCGACCTCTGCATTCTCCCGCGCCGCGCCGGATTTTGGGTACAAATGATCCCAGCAACTTGTATCAATTAAATGCTTTGCTTAGTCTTGGAAACGTCAAAGTGAAACCCCTCCACTGTGGGGATTGTTTCATAAAAGATTTCATTTGAGAGAAGATGGTATAATATTTTGGGTAGCCGTGCAATGACACTAGCCATGGTGACTGGCC

>mPing_5'_3'_A12C

GGCCAGTCACACTGGGGGTTTCACTGGTGTGTCATGCACATTTAATAGGGGTAAGACTGAATAAAAAATGATTATTTGCATGAAATGGGGATGAGAGAGAAGGAAAGAGTTTCATCCTGGTGAAACTCGTCAGCGTCGTTTCCAAGTCCTCGGTAACAGAGTGAAACCCCCGTTGAGGCCGATTCGTTTCATTCACCGGATCTCTTGCGTCCGCCTCCGCCGTGCGACCTCTGCATTCTCCCGCGCCGCGCCGGATTTTGGGTACAAATGATCCCAGCAACTTGTATCAATTAAATGCTTTGCTTAGTCTTGGAAACGTCAAAGTGAAACCCCTCCACTGTGGGGATTGTTTCATAAAAGATTTCATTTGAGAGAAGATGGTATAATATTTTGGGTAGCCGTGCAATGACACTAGCCAGTGTGACTGGCC

>mPing_5'_3'_C3A

GGACAGTCACAATGGGGGTTTCACTGGTGTGTCATGCACATTTAATAGGGGTAAGACTGAATAAAAAATGATTATTTGCATGAAATGGGGATGAGAGAGAAGGAAAGAGTTTCATCCTGGTGAAACTCGTCAGCGTCGTTTCCAAGTCCTCGGTAACAGAGTGAAACCCCCGTTGAGGCCGATTCGTTTCATTCACCGGATCTCTTGCGTCCGCCTCCGCCGTGCGACCTCTGCATTCTCCCGCGCCGCGCCGGATTTTGGGTACAAATGATCCCAGCAACTTGTATCAATTAAATGCTTTGCTTAGTCTTGGAAACGTCAAAGTGAAACCCCTCCACTGTGGGGATTGTTTCATAAAAGATTTCATTTGAGAGAAGATGGTATAATATTTTGGGTAGCCGTGCAATGACACTAGCCATTGTGACTGTCC

>mPing_5'_3'_C4A

GGCAAGTCACAATGGGGGTTTCACTGGTGTGTCATGCACATTTAATAGGGGTAAGACTGAATAAAAAATGATTATTTGCATGAAATGGGGATGAGAGAGAAGGAAAGAGTTTCATCCTGGTGAAACTCGTCAGCGTCGTTTCCAAGTCCTCGGTAACAGAGTGAAACCCCCGTTGAGGCCGATTCGTTTCATTCACCGGATCTCTTGCGTCCGCCTCCGCCGTGCGACCTCTGCATTCTCCCGCGCCGCGCCGGATTTTGGGTACAAATGATCCCAGCAACTTGTATCAATTAAATGCTTTGCTTAGTCTTGGAAACGTCAAAGTGAAACCCCTCCACTGTGGGGATTGTTTCATAAAAGATTTCATTTGAGAGAAGATGGTATAATATTTTGGGTAGCCGTGCAATGACACTAGCCATTGTGACTTGCC

>mPing_5'_3'_C8A

GGCCAGTAACAATGGGGGTTTCACTGGTGTGTCATGCACATTTAATAGGGGTAAGACTGAATAAAAAATGATTATTTGCATGAAATGGGGATGAGAGAGAAGGAAAGAGTTTCATCCTGGTGAAACTCGTCAGCGTCGTTTCCAAGTCCTCGGTAACAGAGTGAAACCCCCGTTGAGGCCGATTCGTTTCATTCACCGGATCTCTTGCGTCCGCCTCCGCCGTGCGACCTCTGCATTCTCCCGCGCCGCGCCGGATTTTGGGTACAAATGATCCCAGCAACTTGTATCAATTAAATGCTTTGCTTAGTCTTGGAAACGTCAAAGTGAAACCCCTCCACTGTGGGGATTGTTTCATAAAAGATTTCATTTGAGAGAAGATGGTATAATATTTTGGGTAGCCGTGCAATGACACTAGCCATTGTTACTGGCC

>mPing_5'_3'_C10A

GGCCAGTCAAAATGGGGGTTTCACTGGTGTGTCATGCACATTTAATAGGGGTAAGACTGAATAAAAAATGATTATTTGCATGAAATGGGGATGAGAGAGAAGGAAAGAGTTTCATCCTGGTGAAACTCGTCAGCGTCGTTTCCAAGTCCTCGGTAACAGAGTGAAACCCCCGTTGAGGCCGATTCGTTTCATTCACCGGATCTCTTGCGTCCGCCTCCGCCGTGCGACCTCTGCATTCTCCCGCGCCGCGCCGGATTTTGGGTACAAATGATCCCAGCAACTTGTATCAATTAAATGCTTTGCTTAGTCTTGGAAACGTCAAAGTGAAACCCCTCCACTGTGGGGATTGTTTCATAAAAGATTTCATTTGAGAGAAGATGGTATAATATTTTGGGTAGCCGTGCAATGACACTAGCCATTTTGACTGGCC

>mPing_5'_3'_G1T

TGCCAGTCACAATGGGGGTTTCACTGGTGTGTCATGCACATTTAATAGGGGTAAGACTGAATAAAAAATGATTATTTGCATGAAATGGGGATGAGAGAGAAGGAAAGAGTTTCATCCTGGTGAAACTCGTCAGCGTCGTTTCCAAGTCCTCGGTAACAGAGTGAAACCCCCGTTGAGGCCGATTCGTTTCATTCACCGGATCTCTTGCGTCCGCCTCCGCCGTGCGACCTCTGCATTCTCCCGCGCCGCGCCGGATTTTGGGTACAAATGATCCCAGCAACTTGTATCAATTAAATGCTTTGCTTAGTCTTGGAAACGTCAAAGTGAAACCCCTCCACTGTGGGGATTGTTTCATAAAAGATTTCATTTGAGAGAAGATGGTATAATATTTTGGGTAGCCGTGCAATGACACTAGCCATTGTGACTGGCA

>mPing_5'_3'_G2T

GTCCAGTCACAATGGGGGTTTCACTGGTGTGTCATGCACATTTAATAGGGGTAAGACTGAATAAAAAATGATTATTTGCATGAAATGGGGATGAGAGAGAAGGAAAGAGTTTCATCCTGGTGAAACTCGTCAGCGTCGTTTCCAAGTCCTCGGTAACAGAGTGAAACCCCCGTTGAGGCCGATTCGTTTCATTCACCGGATCTCTTGCGTCCGCCTCCGCCGTGCGACCTCTGCATTCTCCCGCGCCGCGCCGGATTTTGGGTACAAATGATCCCAGCAACTTGTATCAATTAAATGCTTTGCTTAGTCTTGGAAACGTCAAAGTGAAACCCCTCCACTGTGGGGATTGTTTCATAAAAGATTTCATTTGAGAGAAGATGGTATAATATTTTGGGTAGCCGTGCAATGACACTAGCCATTGTGACTGGAC

>mPing_5'_3'_G6T

GGCCATTCACAATGGGGGTTTCACTGGTGTGTCATGCACATTTAATAGGGGTAAGACTGAATAAAAAATGATTATTTGCATGAAATGGGGATGAGAGAGAAGGAAAGAGTTTCATCCTGGTGAAACTCGTCAGCGTCGTTTCCAAGTCCTCGGTAACAGAGTGAAACCCCCGTTGAGGCCGATTCGTTTCATTCACCGGATCTCTTGCGTCCGCCTCCGCCGTGCGACCTCTGCATTCTCCCGCGCCGCGCCGGATTTTGGGTACAAATGATCCCAGCAACTTGTATCAATTAAATGCTTTGCTTAGTCTTGGAAACGTCAAAGTGAAACCCCTCCACTGTGGGGATTGTTTCATAAAAGATTTCATTTGAGAGAAGATGGTATAATATTTTGGGTAGCCGTGCAATGACACTAGCCATTGTGAATGGCC

>mPing_5'_3'_G14T

GGCCAGTCACAATTGGGGTTTCACTGGTGTGTCATGCACATTTAATAGGGGTAAGACTGAATAAAAAATGATTATTTGCATGAAATGGGGATGAGAGAGAAGGAAAGAGTTTCATCCTGGTGAAACTCGTCAGCGTCGTTTCCAAGTCCTCGGTAACAGAGTGAAACCCCCGTTGAGGCCGATTCGTTTCATTCACCGGATCTCTTGCGTCCGCCTCCGCCGTGCGACCTCTGCATTCTCCCGCGCCGCGCCGGATTTTGGGTACAAATGATCCCAGCAACTTGTATCAATTAAATGCTTTGCTTAGTCTTGGAAACGTCAAAGTGAAACCCCTCCACTGTGGGGATTGTTTCATAAAAGATTTCATTTGAGAGAAGATGGTATAATATTTTGGGTAGCCGTGCAATGACACTAGCAATTGTGACTGGCC

>mPing_5'_3'_G15T

GGCCAGTCACAATGTGGGTTTCACTGGTGTGTCATGCACATTTAATAGGGGTAAGACTGAATAAAAAATGATTATTTGCATGAAATGGGGATGAGAGAGAAGGAAAGAGTTTCATCCTGGTGAAACTCGTCAGCGTCGTTTCCAAGTCCTCGGTAACAGAGTGAAACCCCCGTTGAGGCCGATTCGTTTCATTCACCGGATCTCTTGCGTCCGCCTCCGCCGTGCGACCTCTGCATTCTCCCGCGCCGCGCCGGATTTTGGGTACAAATGATCCCAGCAACTTGTATCAATTAAATGCTTTGCTTAGTCTTGGAAACGTCAAAGTGAAACCCCTCCACTGTGGGGATTGTTTCATAAAAGATTTCATTTGAGAGAAGATGGTATAATATTTTGGGTAGCCGTGCAATGACACTAGACATTGTGACTGGCC

>mPing_5'_3'_T7G

GGCCAGGCACAATGGGGGTTTCACTGGTGTGTCATGCACATTTAATAGGGGTAAGACTGAATAAAAAATGATTATTTGCATGAAATGGGGATGAGAGAGAAGGAAAGAGTTTCATCCTGGTGAAACTCGTCAGCGTCGTTTCCAAGTCCTCGGTAACAGAGTGAAACCCCCGTTGAGGCCGATTCGTTTCATTCACCGGATCTCTTGCGTCCGCCTCCGCCGTGCGACCTCTGCATTCTCCCGCGCCGCGCCGGATTTTGGGTACAAATGATCCCAGCAACTTGTATCAATTAAATGCTTTGCTTAGTCTTGGAAACGTCAAAGTGAAACCCCTCCACTGTGGGGATTGTTTCATAAAAGATTTCATTTGAGAGAAGATGGTATAATATTTTGGGTAGCCGTGCAATGACACTAGCCATTGTGCCTGGCC

>mPing_5'_3'_T13G

GGCCAGTCACAAGGGGGGTTTCACTGGTGTGTCATGCACATTTAATAGGGGTAAGACTGAATAAAAAATGATTATTTGCATGAAATGGGGATGAGAGAGAAGGAAAGAGTTTCATCCTGGTGAAACTCGTCAGCGTCGTTTCCAAGTCCTCGGTAACAGAGTGAAACCCCCGTTGAGGCCGATTCGTTTCATTCACCGGATCTCTTGCGTCCGCCTCCGCCGTGCGACCTCTGCATTCTCCCGCGCCGCGCCGGATTTTGGGTACAAATGATCCCAGCAACTTGTATCAATTAAATGCTTTGCTTAGTCTTGGAAACGTCAAAGTGAAACCCCTCCACTGTGGGGATTGTTTCATAAAAGATTTCATTTGAGAGAAGATGGTATAATATTTTGGGTAGCCGTGCAATGACACTAGCCCTTGTGACTGGCC

>mPing_5'_C3A

GGACAGTCACAATGGGGGTTTCACTGGTGTGTCATGCACATTTAATAGGGGTAAGACTGAATAAAAAATGATTATTTGCATGAAATGGGGATGAGAGAGAAGGAAAGAGTTTCATCCTGGTGAAACTCGTCAGCGTCGTTTCCAAGTCCTCGGTAACAGAGTGAAACCCCCGTTGAGGCCGATTCGTTTCATTCACCGGATCTCTTGCGTCCGCCTCCGCCGTGCGACCTCTGCATTCTCCCGCGCCGCGCCGGATTTTGGGTACAAATGATCCCAGCAACTTGTATCAATTAAATGCTTTGCTTAGTCTTGGAAACGTCAAAGTGAAACCCCTCCACTGTGGGGATTGTTTCATAAAAGATTTCATTTGAGAGAAGATGGTATAATATTTTGGGTAGCCGTGCAATGACACTAGCCATTGTGACTGGCC

>mPing_5'_G1T

TGCCAGTCACAATGGGGGTTTCACTGGTGTGTCATGCACATTTAATAGGGGTAAGACTGAATAAAAAATGATTATTTGCATGAAATGGGGATGAGAGAGAAGGAAAGAGTTTCATCCTGGTGAAACTCGTCAGCGTCGTTTCCAAGTCCTCGGTAACAGAGTGAAACCCCCGTTGAGGCCGATTCGTTTCATTCACCGGATCTCTTGCGTCCGCCTCCGCCGTGCGACCTCTGCATTCTCCCGCGCCGCGCCGGATTTTGGGTACAAATGATCCCAGCAACTTGTATCAATTAAATGCTTTGCTTAGTCTTGGAAACGTCAAAGTGAAACCCCTCCACTGTGGGGATTGTTTCATAAAAGATTTCATTTGAGAGAAGATGGTATAATATTTTGGGTAGCCGTGCAATGACACTAGCCATTGTGACTGGCC

>mPing_5'_G2T

GTCCAGTCACAATGGGGGTTTCACTGGTGTGTCATGCACATTTAATAGGGGTAAGACTGAATAAAAAATGATTATTTGCATGAAATGGGGATGAGAGAGAAGGAAAGAGTTTCATCCTGGTGAAACTCGTCAGCGTCGTTTCCAAGTCCTCGGTAACAGAGTGAAACCCCCGTTGAGGCCGATTCGTTTCATTCACCGGATCTCTTGCGTCCGCCTCCGCCGTGCGACCTCTGCATTCTCCCGCGCCGCGCCGGATTTTGGGTACAAATGATCCCAGCAACTTGTATCAATTAAATGCTTTGCTTAGTCTTGGAAACGTCAAAGTGAAACCCCTCCACTGTGGGGATTGTTTCATAAAAGATTTCATTTGAGAGAAGATGGTATAATATTTTGGGTAGCCGTGCAATGACACTAGCCATTGTGACTGGCC

>mPing_90_mPong

GGCCAGTCACAATGGGGGTTTCACTGGTGTGTCATGCACATTTAATAGGGGTAAGACTGAATAAAAAATGATTATTTGCATGAAATGGGGAGGAGAGAGAGTAAACTCGTTTCACCATGGTGACACGAGATAGCGCCGTTTCCCAGGTCGCTGAAACGGGGTGAAACAGCATTGAGAGTTCATCGTTTCACCTCCGGGATCCCGTGCGAGCGCTGCTCTTCGCCATCTTCGCGCGCATCGCCGGATTCTTCCACATACGATCCCACTATGTGGCTGGAATTAAATGCCTTGAATTTGCATTGGAAACGCTAGAGTGAAACACAGCATTGAGAAGGTCTGTTGGGGATTGTTTCATAAAAGATTTCATTTGAGAGAAGATGGTATAATATTTTGGGTAGCCGTGCAATGACACTAGCCATTGTGACTGGCC

>mPing/mPong_half

GGCCAGTCACAATGGGGGTTTCACTGGTGTGTCATGCACATTTAATAGGGGTAAGACTGAATAAAAAATGATTATTTGCATGAAATGGGGATGAGAGAGAAGGAAAGAGTTTCATCCTGGTGAAACTCGTCAGCGTCGTTTCCAAGTCCTCGGTAACAGAGTGAAACCCCCGTTGAGGCCGATTCGTTTCATTCACCGGATCTCTTGCGTCCGCCCTCTTCGCCATCTTCGCGCGCATCGCCGGATTCTTCCACATACGATCCCACTATGTGGCTGGAATTAAATGCCTTGAATTTGCATTGGAAACGCTAGAGTGAAACACAGCATTGAGAAGGTCTGTTTCATTGTACGTTTCAACTTGTTTCATCTTCGTTTCAGCTGATGTGGCGTCTGGGAAACAGTGTAATGAAACACTGCATTGTGAATGGCC

>mPong

GGCCAGTCACAATGGGTGTTTCATTTGAGTGTCATGCGCATTTAATACAGTGACAAGTCAGCAAAAGAGCAATATTTGCATGAAATGGGTAGGAGAGAGAGTAAACTCGTTTCACCATGGTGACACGAGATAGCGCCGTTTCCCAGGTCGCTGAAACGGGGTGAAACAGCATTGAGAGTTCATCGTTTCACCTCCGGGATCCCGTGCGAGCGCTGCTCTTCGCCATCTTCGCGCGCATCGCCGGATTCTTCCACATACGATCCCACTATGTGGCTGGAATTAAATGCCTTGAATTTGCATTGGAAACGCTAGAGTGAAACACAGCATTGAGAAGGTCTGTTTCATTGTACGTTTCAACTTGTTTCATCTTCGTTTCAGCTGATGTGGCGTCTGGGAAACAGTGTAATGAAACACTGCATTGTGAATGGCC

>mPong_90_mPing

GGCCAGTCACAATGGGTGTTTCATTTGAGTGTCATGCGCATTTAATACAGTGACAAGTCAGCAAAAGAGCAATATTTGCATGAAATGGGTATGAGAGAGAAGGAAAGAGTTTCATCCTGGTGAAACTCGTCAGCGTCGTTTCCAAGTCCTCGGTAACAGAGTGAAACCCCCGTTGAGGCCGATTCGTTTCATTCACCGGATCTCTTGCGTCCGCCTCCGCCGTGCGACCTCTGCATTCTCCCGCGCCGCGCCGGATTTTGGGTACAAATGATCCCAGCAACTTGTATCAATTAAATGCTTTGCTTAGTCTTGGAAACGTCAAAGTGAAACCCCTCCACTGTTCATTGTACGTTTCAACTTGTTTCATCTTCGTTTCAGCTGATGTGGCGTCTGGGAAACAGTGTAATGAAACACTGCATTGTGAATGGCC

>mPong/mPing_half

GGCCAGTCACAATGGGTGTTTCATTTGAGTGTCATGCGCATTTAATACAGTGACAAGTCAGCAAAAGAGCAATATTTGCATGAAATGGGTAGGAGAGAGAGTAAACTCGTTTCACCATGGTGACACGAGATAGCGCCGTTTCCCAGGTCGCTGAAACGGGGTGAAACAGCATTGAGAGTTCATCGTTTCACCTCCGGGATCCCGTGCGAGCGCTGTCCGCCGTGCGACCTCTGCATTCTCCCGCGCCGCGCCGGATTTTGGGTACAAATGATCCCAGCAACTTGTATCAATTAAATGCTTTGCTTAGTCTTGGAAACGTCAAAGTGAAACCCCTCCACTGTGGGGATTGTTTCATAAAAGATTTCATTTGAGAGAAGATGGTATAATATTTTGGGTAGCCGTGCAATGACACTAGCCATTGTGACTGGCC

>Ping16A

GGCCAGTCACAATGGAGGTTTCACTGGTGTGTCATGCACATTTAATAGGGGTAAGACTGAATAAAAAATGATTATTTGCATGAAATGGGGATGAGAGAGAAGGAAAGAGTTTCATCCTGGTGAAACTCGTCAGCGTCGTTTCCAAGTCCTCGGTAACAGAGTGAAACCCCCGTTGAGGCCGATTCGTTTCATTCACCGGATCTCTTGCGTCCGCCTCCGCCGTGCGACCTCCGCATTCTCCCGCGCCGCGCCGCGCCACGCCTCCTTCCCGCGTGAACATTCCTCCTTCCCGCGCGAGCGATTCCACCATCTCCCCCGTCCGGCGCCTACGGAGTACACCGCAACCGGTCGCCCCAATCCGGCGCCTAGACCGTGACCCACCCGCCATCTTCCGCAAGACCGAATCCCCAACCCACCCACCATCTTCCGCCGCCCCCGTCCCCGTCCCCGGCCATGGATCCGTCGCCGGCCGTGGATCCGTCGCCGGCCGTGGATCCGTCGCCGGCTGCTGAAACCCGGCGGCGTGCAACCGGGAAAGGAGGCAAACAGCGCGGGGGCAAGCAACTAGGATTGAAGAGGCCGCCGCCGATTTCTGTCCCGGCCACCCCGCCTCCTGCTGCGACGTCTTCATCCCCTGCTGCGCCGACGGCCATCCCACCACGACCACCGCAATCTTCGCCGATTTTCGTCCCCGATTCGCCGAATCCGTCACCGGCTGCGCCGACCTCCTCTCTTGCTTCGGGGACATCGACGGCAAGGCCACCGCAACCACAAGGAGGAGGATGGGGACCAACATCGACCATTTCCCCAAACTTTGCATCTTTCTTTGGAAACCAACAAGACCCAAATTCATGGTACATGTATTTTCTTCTTTTTCTGTTACTTTCAACCTACGGTAACTCTAATTCATGGATGAGACTACTGCCATTGTGCAGTTCAATGCTTTTTCTTCATGTTATATTTCGTCCAGCTGTGAGTTATGGTTTGAAGATTGCTGTGGTTGTTTCATTGCTGAGTATGTGAAAGATAGATGGATGAAAGAGAGAATTATATTTTAGTCTGTAATCTTGCTCATCCAGTTGCTCATGTATGACCTTGGTTCTAGAATGTTGCCCTGACTGTATGCTTAATGTTCAGAGAAGTGATGCCTAAAGCAGTGAGATCAGTGGGATCAGATTAGCTATCGACATATAATATTAGCTATCTCAGTTGTGAAAGAGAGATGGGTGAAAAGGCACCCCTTGGATTAATTCTGTAGTATCAAATTCTGCACCTTGTCTGTCCATATGTTCTGCTTGGTTGGTGGGTGCAGTGCATTTGTAAAAAATAGTTTGCTTCTGATCCTTAATATATGTAACAGGGAATGAATTTTCACCCATCTCAGTTGTAAAGGTACTGTCTTGCTATGCAATATGTGTAAATTGACAAACCTGAAAATAGTCTGTTTGGAATTTGCAAAAGCAATTCGATAGTTTGGAATTTCCAAACCTCAGTCAGCAGTAGGCAATCCATTTTAGTTCTTGCTATGCACAAAAACAGTACACCTGATATGCTCATTTTAATACAACTTTTTTGTCTCTGTTACAGTTTGGTCAGGGGTTATCCTCCAGGAGGGTTTGTCAATTTTATTCAACAAAATTGTCCGCCGCAGCCACAACAGCAAGGTGAAAATTTTCATTTCGTTGGTCACAATATGGGATTCAACCCAATATCTCCACAGCCACCAAGTGCCTACGGAACACCAACACCCCAAGCTACGAACCAAGGCACTTCAACAAACATTATGATTGATGAAGAGGACAACAATGATGACAGTAGGGCAGCAAAGAAAAGATGGACTCATGAAGAGGAAGAGAGACTGGTATTCATCGGATACTTTTACATTTCCATATGTCTTTGTTTTGACTAATACTTGACAGGTCATTAACTGATTCTTGTAGGCCAGTGCTTGGTTGAATGCTTCTAAAGACTCAATTCATGGGAATGATAAGAAAGGTGATACATTTTGGAAGGAAGTCACTGATGAATTTAACAAGAAAGGGAATGGAAAACGTAGGAGGGAAATTAACCAACTGAAGGTTCACTGGTCAAGGTTGAAGTCAGCGATCTCTGAGTTCAATGACTATTGGAGTACGGTTACTCAAATGCATACAAGCGGATACTCCGACGACATGCTTGAGAAAGAGGCACAGAGGCTGTATGCAAACAGGTTTGGAAAACCTTTTGCGTTGGTCCATTGGTGGAAGATACTCAAAGATGAGCCCAAATGGTGTGCTCAGTTTGAATCAGAGAAAGACAAGAGCGAAATGGATGCTGTTCCAGAACAGCAGTCACGTCCTATTGGTAGAGAAGCAGCAAAGTCTGAGCGCAATGGAAAGCGCAAGAAAGAAAATGTTATGGAAGGCATTGTCCTCCTAGGGGACAATGTCCAGAAAATTATAAAGGTCCACGAAGACCGGAGGGTGGATCGTGAAAAGGCCACCGAAGCACAGATTCAGATATCAAATGCAACATTGTTGGCCGCTAAGGAGCAGAAGGAAGCAAAGATGTTCGATGTGTACAATACTCTATTAAGTAAGGATACAAGCAACATGTCTGAAGATCAAATGGCTAGCCACCAGAGGGCAATACGGAAATTAGAGGAGAAGCTATTTGCGGATTAAGGTGAGTTTTATAAACTGACCACTATTTTCTGAAATGTATGAATTCTGAAATTTATATACAATTGTGTAAACATGGAAAATTAGATAATGTATGCATGATGCACAACATGTGCGTGCAGCACTATTTAATGGCAGTTTCACAAGTGTGAAAACTGACCACTATAGTACTATTGTGGTGTGAAAACTGACCACTACTATTGTGGTGTGAATGCTACTGTGGTGTGAAAACTGACCACTATAGTTTCACATTCCTGGATGCAGCCCTCCTCTATATATATAGATACAGTCCTCATCTCTTCCTGGCATACACACAGCCCTCTTCTCTAATTCCTGGACGCAGTCCTCATCTCTTCCTGGCATAGACGCAGCCCTTCTCTCTTCCTGTTTAGTTCAACAACATTGAGGTGATCTGCCTTTCTTTGAAGTTTCTATCTTTTTTCACTGCTGTGAATGATTATTTCTCTGCTGTGAATGATTATTTCTCCAATCTTCCTTTGTTCACCTTCTCTCTTTCTCTGCTGTGAAGATGTCTGGAAATGAAAATCAGATTCCTGTGTCCTTGTTGGACGAGTTTCTCGCTGAGGATGAGATCATGGATGAGATAATGGATGATGTTCTCCATGAAATGATGGTGTTATTGCAGTCCTCCATCGGAGATCTTGAAAGAGAGGCTGCTGACCATCGTTTGCATCCAAGGAAGCACATCAAGAGGCCACGAGAGGAAGCACATCAAAATTTGGTGAATGATTATTTCTCTGAAAATCCTCTATATCCTTCCAATATTTTTCGCCGAAGATTTCGTATGTACAGGCCGCTGTTTTTACGTATTGTGGACGCATTAGGCCAGTGGTCAGATTACTTTACTCAGAGGGTAGATGCCGCTGGTAGGCAAGGGCTTAGTCCATTACAAAAGTGTACTGCAGCAATTCGCCAATTGGCTACTGGTAGTGGTGCTGATGAACTAGATGAGTATTTGAAGATTGGAGAGACTACTGCTATGGATGCTATGAAAAATTTTGTGAAAGGAATTAGAGAAGTATTTGGTGAAAGATATCTCAGGCGTCCCACTGTAGAAGATACTGAACGACTACTCGAGCTTGGTGAGAGACGCGGTTTTCCTGGTATGTTCGGTAGCATTGACTGTATGCATTGGCAATGGGAAAGGTGCCCAACTGCGTGGAAGGGTCAGTTCACTCGTGGTGATCAAAAAGTGCCAACGCTGATTCTTGAGGCAGTGGCATCACATGATCTTTGGATTTGGCATGCGTTCTTTGGAGTAGCAGGTTCTAACAATGATATCAATGTTTTGAGCCGATCTACTGTGTTTATCAATGAGCTGAAAGGACAAGCTCCTAGAGTGCAGTACATGGTAAATGGGAATCAATACAACGAAGGTTATTTTCTTGCTGATGGAATTTACCCTGAATGGAAGGTATTTGCTAAGTCATATCGACTCCCTATCACTGAGAAGGAGAAGTTGTATGCACAACATCAAGAAGGGGCAAGAAAGGATATCGAGAGAGCATTTGGTGTTCTACAACGTCGATTCTGCATCTTAAAACGACCAGCCCGTCTATATGACCGAGGTGTACTCCGTGATGTTGTCCTAGGTTGCATCATACTTCACAATATGATAGTTGAAGATGAGAAGGAAGCGCGACTTATTGAAGAAAATCTAGATTTAAATGAGCCTGCTAGTTCATCAACGGTTCAGGCACCAGAATTCTCTCCTGACCAGCATGTTCCATTAGAAAGAATTTTAGAAAAGGATACTAGTATGAGAGATCGTTTGGCTCATCGCCGACTCAAGAATGATTTGGTGGAACATATATGGAATAAGTTTGGTGGTGGTGCACATTCATCTGGTAATTATGTTTTTATTTTGCATTATTAGTTATCTATGGTACTAAGATATGTACAAGTTTCTCTAAATTGCACTAAATCTGTGGTTCATATTGGATATGTGTAAACTATGAATGTAGCCTGACTAAAACCATCATTCATGCTGAACTGGTTTTTGTTTTGTATATGCAGGATGAAACAAGGAACTAGGTTTCTGAACGCATTACGGACTGAAGGTTGAGGGGCAGAATGATCCACCCAGTTGCTTCTATCAGATCACTAAAGTTTCATTTCACTGTTTTATTTTGGACACTTGATGCTTGTGTGCATCCGATGAATGTTTAATTTGGTCACCTGATGCTTGTGTGCATCCGATGAATGTTTAATTTGGTCACCTGATGCTTGTATGCAGTTATCTATCTTATTTGTTAATGTTGCTGGTACTGAGGATTTTTAGAAGTGAAATGCACAAGTTGCTGTGTTTTTTGACTGATCCTTGTGTGCACTTGACGTTGTATGTGACAAATGATGGTTCCCAGTTGTGCACCTGATTCATGATTCAGTTATTCAGTTTAAATTGACGTTGTTTGTGTGCACCTTTTGTCAGTTAGCCAGTTACGGCTGGAAGTTGTGTAAGTTTGTGTGACGCCTGGCTACAGGATTTTGGGTACAAATGATCCCAGCAACTTGTATCAATTAAATGCTTTGCTTAGTCTTGGAAACGTCAAAGTGAAACCCCTCCACTGTGGGGATTGTTTCATAAAAGATTTCATTTGAGAGAAGATGGTATAATATTTTGGGTAGCCGTGCAATGACACTAGCCATTGTGACTGGCC

>Ping16G

GGCCAGTCACAATGGGGGTTTCACTGGTGTGTCATGCACATTTAATAGGGGTAAGACTGAATAAAAAATGATTATTTGCATGAAATGGGGATGAGAGAGAAGGAAAGAGTTTCATCCTGGTGAAACTCGTCAGCGTCGTTTCCAAGTCCTCGGTAACAGAGTGAAACCCCCGTTGAGGCCGATTCGTTTCATTCACCGGATCTCTTGCGTCCGCCTCCGCCGTGCGACCTCCGCATTCTCCCGCGCCGCGCCGCGCCACGCCTCCTTCCCGCGTGAACATTCCTCCTTCCCGCGCGAGCGATTCCACCATCTCCCCCGTCCGGCGCCTACGGAGTACACCGCAACCGGTCGCCCCAATCCGGCGCCTAGACCGTGACCCACCCGCCATCTTCCGCAAGACCGAATCCCCAACCCACCCACCATCTTCCGCCGCCCCCGTCCCCGTCCCCGGCCATGGATCCGTCGCCGGCCGTGGATCCGTCGCCGGCCGTGGATCCGTCGCCGGCTGCTGAAACCCGGCGGCGTGCAACCGGGAAAGGAGGCAAACAGCGCGGGGGCAAGCAACTAGGATTGAAGAGGCCGCCGCCGATTTCTGTCCCGGCCACCCCGCCTCCTGCTGCGACGTCTTCATCCCCTGCTGCGCCGACGGCCATCCCACCACGACCACCGCAATCTTCGCCGATTTTCGTCCCCGATTCGCCGAATCCGTCACCGGCTGCGCCGACCTCCTCTCTTGCTTCGGGGACATCGACGGCAAGGCCACCGCAACCACAAGGAGGAGGATGGGGACCAACATCGACCATTTCCCCAAACTTTGCATCTTTCTTTGGAAACCAACAAGACCCAAATTCATGGTACATGTATTTTCTTCTTTTTCTGTTACTTTCAACCTACGGTAACTCTAATTCATGGATGAGACTACTGCCATTGTGCAGTTCAATGCTTTTTCTTCATGTTATATTTCGTCCAGCTGTGAGTTATGGTTTGAAGATTGCTGTGGTTGTTTCATTGCTGAGTATGTGAAAGATAGATGGATGAAAGAGAGAATTATATTTTAGTCTGTAATCTTGCTCATCCAGTTGCTCATGTATGACCTTGGTTCTAGAATGTTGCCCTGACTGTATGCTTAATGTTCAGAGAAGTGATGCCTAAAGCAGTGAGATCAGTGGGATCAGATTAGCTATCGACATATAATATTAGCTATCTCAGTTGTGAAAGAGAGATGGGTGAAAAGGCACCCCTTGGATTAATTCTGTAGTATCAAATTCTGCACCTTGTCTGTCCATATGTTCTGCTTGGTTGGTGGGTGCAGTGCATTTGTAAAAAATAGTTTGCTTCTGATCCTTAATATATGTAACAGGGAATGAATTTTCACCCATCTCAGTTGTAAAGGTACTGTCTTGCTATGCAATATGTGTAAATTGACAAACCTGAAAATAGTCTGTTTGGAATTTGCAAAAGCAATTCGATAGTTTGGAATTTCCAAACCTCAGTCAGCAGTAGGCAATCCATTTTAGTTCTTGCTATGCACAAAAACAGTACACCTGATATGCTCATTTTAATACAACTTTTTTGTCTCTGTTACAGTTTGGTCAGGGGTTATCCTCCAGGAGGGTTTGTCAATTTTATTCAACAAAATTGTCCGCCGCAGCCACAACAGCAAGGTGAAAATTTTCATTTCGTTGGTCACAATATGGGATTCAACCCAATATCTCCACAGCCACCAAGTGCCTACGGAACACCAACACCCCAAGCTACGAACCAAGGCACTTCAACAAACATTATGATTGATGAAGAGGACAACAATGATGACAGTAGGGCAGCAAAGAAAAGATGGACTCATGAAGAGGAAGAGAGACTGGTATTCATCGGATACTTTTACATTTCCATATGTCTTTGTTTTGACTAATACTTGACAGGTCATTAACTGATTCTTGTAGGCCAGTGCTTGGTTGAATGCTTCTAAAGACTCAATTCATGGGAATGATAAGAAAGGTGATACATTTTGGAAGGAAGTCACTGATGAATTTAACAAGAAAGGGAATGGAAAACGTAGGAGGGAAATTAACCAACTGAAGGTTCACTGGTCAAGGTTGAAGTCAGCGATCTCTGAGTTCAATGACTATTGGAGTACGGTTACTCAAATGCATACAAGCGGATACTCCGACGACATGCTTGAGAAAGAGGCACAGAGGCTGTATGCAAACAGGTTTGGAAAACCTTTTGCGTTGGTCCATTGGTGGAAGATACTCAAAGATGAGCCCAAATGGTGTGCTCAGTTTGAATCAGAGAAAGACAAGAGCGAAATGGATGCTGTTCCAGAACAGCAGTCACGTCCTATTGGTAGAGAAGCAGCAAAGTCTGAGCGCAATGGAAAGCGCAAGAAAGAAAATGTTATGGAAGGCATTGTCCTCCTAGGGGACAATGTCCAGAAAATTATAAAGGTCCACGAAGACCGGAGGGTGGATCGTGAAAAGGCCACCGAAGCACAGATTCAGATATCAAATGCAACATTGTTGGCCGCTAAGGAGCAGAAGGAAGCAAAGATGTTCGATGTGTACAATACTCTATTAAGTAAGGATACAAGCAACATGTCTGAAGATCAAATGGCTAGCCACCAGAGGGCAATACGGAAATTAGAGGAGAAGCTATTTGCGGATTAAGGTGAGTTTTATAAACTGACCACTATTTTCTGAAATGTATGAATTCTGAAATTTATATACAATTGTGTAAACATGGAAAATTAGATAATGTATGCATGATGCACAACATGTGCGTGCAGCACTATTTAATGGCAGTTTCACAAGTGTGAAAACTGACCACTATAGTACTATTGTGGTGTGAAAACTGACCACTACTATTGTGGTGTGAATGCTACTGTGGTGTGAAAACTGACCACTATAGTTTCACATTCCTGGATGCAGCCCTCCTCTATATATATAGATACAGTCCTCATCTCTTCCTGGCATACACACAGCCCTCTTCTCTAATTCCTGGACGCAGTCCTCATCTCTTCCTGGCATAGACGCAGCCCTTCTCTCTTCCTGTTTAGTTCAACAACATTGAGGTGATCTGCCTTTCTTTGAAGTTTCTATCTTTTTTCACTGCTGTGAATGATTATTTCTCTGCTGTGAATGATTATTTCTCCAATCTTCCTTTGTTCACCTTCTCTCTTTCTCTGCTGTGAAGATGTCTGGAAATGAAAATCAGATTCCTGTGTCCTTGTTGGACGAGTTTCTCGCTGAGGATGAGATCATGGATGAGATAATGGATGATGTTCTCCATGAAATGATGGTGTTATTGCAGTCCTCCATCGGAGATCTTGAAAGAGAGGCTGCTGACCATCGTTTGCATCCAAGGAAGCACATCAAGAGGCCACGAGAGGAAGCACATCAAAATTTGGTGAATGATTATTTCTCTGAAAATCCTCTATATCCTTCCAATATTTTTCGCCGAAGATTTCGTATGTACAGGCCGCTGTTTTTACGTATTGTGGACGCATTAGGCCAGTGGTCAGATTACTTTACTCAGAGGGTAGATGCCGCTGGTAGGCAAGGGCTTAGTCCATTACAAAAGTGTACTGCAGCAATTCGCCAATTGGCTACTGGTAGTGGTGCTGATGAACTAGATGAGTATTTGAAGATTGGAGAGACTACTGCTATGGATGCTATGAAAAATTTTGTGAAAGGAATTAGAGAAGTATTTGGTGAAAGATATCTCAGGCGTCCCACTGTAGAAGATACTGAACGACTACTCGAGCTTGGTGAGAGACGCGGTTTTCCTGGTATGTTCGGTAGCATTGACTGTATGCATTGGCAATGGGAAAGGTGCCCAACTGCGTGGAAGGGTCAGTTCACTCGTGGTGATCAAAAAGTGCCAACGCTGATTCTTGAGGCAGTGGCATCACATGATCTTTGGATTTGGCATGCGTTCTTTGGAGTAGCAGGTTCTAACAATGATATCAATGTTTTGAGCCGATCTACTGTGTTTATCAATGAGCTGAAAGGACAAGCTCCTAGAGTGCAGTACATGGTAAATGGGAATCAATACAACGAAGGTTATTTTCTTGCTGATGGAATTTACCCTGAATGGAAGGTATTTGCTAAGTCATATCGACTCCCTATCACTGAGAAGGAGAAGTTGTATGCACAACATCAAGAAGGGGCAAGAAAGGATATCGAGAGAGCATTTGGTGTTCTACAACGTCGATTCTGCATCTTAAAACGACCAGCCCGTCTATATGACCGAGGTGTACTCCGTGATGTTGTCCTAGGTTGCATCATACTTCACAATATGATAGTTGAAGATGAGAAGGAAGCGCGACTTATTGAAGAAAATCTAGATTTAAATGAGCCTGCTAGTTCATCAACGGTTCAGGCACCAGAATTCTCTCCTGACCAGCATGTTCCATTAGAAAGAATTTTAGAAAAGGATACTAGTATGAGAGATCGTTTGGCTCATCGCCGACTCAAGAATGATTTGGTGGAACATATATGGAATAAGTTTGGTGGTGGTGCACATTCATCTGGTAATTATGTTTTTATTTTGCATTATTAGTTATCTATGGTACTAAGATATGTACAAGTTTCTCTAAATTGCACTAAATCTGTGGTTCATATTGGATATGTGTAAACTATGAATGTAGCCTGACTAAAACCATCATTCATGCTGAACTGGTTTTTGTTTTGTATATGCAGGATGAAACAAGGAACTAGGTTTCTGAACGCATTACGGACTGAAGGTTGAGGGGCAGAATGATCCACCCAGTTGCTTCTATCAGATCACTAAAGTTTCATTTCACTGTTTTATTTTGGACACTTGATGCTTGTGTGCATCCGATGAATGTTTAATTTGGTCACCTGATGCTTGTGTGCATCCGATGAATGTTTAATTTGGTCACCTGATGCTTGTATGCAGTTATCTATCTTATTTGTTAATGTTGCTGGTACTGAGGATTTTTAGAAGTGAAATGCACAAGTTGCTGTGTTTTTTGACTGATCCTTGTGTGCACTTGACGTTGTATGTGACAAATGATGGTTCCCAGTTGTGCACCTGATTCATGATTCAGTTATTCAGTTTAAATTGACGTTGTTTGTGTGCACCTTTTGTCAGTTAGCCAGTTACGGCTGGAAGTTGTGTAAGTTTGTGTGACGCCTGGCTACAGGATTTTGGGTACAAATGATCCCAGCAACTTGTATCAATTAAATGCTTTGCTTAGTCTTGGAAACGTCAAAGTGAAACCCCTCCACTGTGGGGATTGTTTCATAAAAGATTTCATTTGAGAGAAGATGGTATAATATTTTGGGTAGCCGTGCAATGACACTAGCCATTGTGACTGGCC

>Pong

GGCCAGTCACAATGGGTGTTTCATTTGAGTGTCATGCGCATTTAATACAGTGACAAGTCAGCAAAAGAGCAATATTTGCATGAAATGGGTAGGAGAGAGAGTAAACTCGTTTCACCATGGTGACACGAGATAGCGCCGTTTCCCAGGTCACTGAAACGGGGTGAAACAGCATTGAGAGTTCATCGTTTCACCTCCGGGATCCCGTGCGAGCGCTGCTCTTCGCCATCTTCGCGCGCATCGCCGGATTCTTCCCGCGCGAGTCCCCCATCTTCCCGCGCAGCACCTCCATGTTCCCGCCCCCAAAGCACTGGCTCGAAGCTTTTTTCCCCAATCTCACCTGCAACCCTAGCGCCAGACTCAGTCCCCATCGCCCCGTCCGTCCCATACCCTAGCGCAAGAACCACGAGCGGAGATTGCGGAGCTGGATCCACAAGTAGGTGGTGAATCCTGTCCATCTGCCGCCGTCCGCCGTCCAGCAGCCATGGATCCACAAGGAGGTGGTGGATCCCGTCTGAGCGCCGCCGGCAGAGGAGGGAATAAGCGTGGGGGCAAGCAGCTGGGCCTGAAGAGGTCGTCGGCGCCTGCTCCATCACCGGCAACAGCTCAGCCACCGCTGCCTGCAAGTTCCCCTCCTGAAGCTCCATCGCCGGCAACAGTTCAGCCGCCTACTCCATCGTCAAGTCCTGCTGTTGCTGCCCCCAGTTCATCCCCTGCTGTACCGATGTCAACCATGCCCCCATGGCCACCGCAAGGAGCAGGATGGGGCTCTGTACCCCCCAATTTTGCTTTTCTGCAAGGAAACCAACAAGGCCCAAGTTCATGGTATTTTCTCCTTGTCACAGATTATTCATTGTACACTATGATACATGATATGACTCTCTTCTTCATGCATTAGTAATTAGTTCCTGTTTATGCTCAATGAAATTTGTTAGAATCAGTATGTCAGTACATTGGTAATTTGATATATGCCTGAGTAATGAATAGAAAAAATGTAGTATTCAGTATGGATTGCAGTAATACTTTGTTAGTGAAAATTCAGTATTCAGTATGCAGTATGGATTGCGGCTTGTATAACAGAAATTGAAAGCAAAAGATTCAGTTTGCAATCTGGACAGTGTACTGTACAACATGTAATTCACATACGTAAAGCTTGTTAAATATCTCCTTGTCAGTACATTGGTAACAAATGCTTTGAGTGTAAATGCCAAGGGTATCATCCTAACATTGGTATATATTTTTAGCCTTCTGTATGGAATGCAGACATGGTCTTCTTTGCAACCACAGCAACAGCTTGCCCTACACTCTGTGCTGTCGTCATAGCTAACCAAATAACCTGTTAGTACTGATATATATGGTCTTCTTTGCAACCACAGCAACAGCTTGCCCTACATGGTCTTCTGTATGCTTGACTAAACTTGTTACTTGACATATATGCTTGACTGAACTTGTTGCTTGACTGAATTATTCCTTACACATACTGTAGTACTTGCTTGACTGAACTATGTCAGGATCTTATTAAAAAAAATCTATGTCAGCACTGCTACTATGTCAGGATCATCAGTATGATGCTTAAGTAACCTGTTAGTATGTCAGTACTTACTATGTCAGGATCATCTTCTGGAACTTACTATGTTTGATTTTCTTATGCTGCCATCGGTTTCAATTGGATTTGCTTCTTATGTTTTCAGGTTGTATCCTACAGAAGGCTTCGTAAATTTTCTCCAACAGAACTGTCTGCCGCAGCCACAAGAAGGTGAAAATTTTCACCTTGTTGGTCAGACTACCAACACAATGTCTACTCCACCACCAACACCCCAAGCTGCAGCTAACAATACAGTCCAAATTGATATTCATGAAGATGCAATCAATGATGCAAGTGCTAAAAAGAGAAGTTTGAGATATTGGACTCATGATGAGGAAGAGAGATTGGCTAGTGCTTGGTTGAATGCTTCTAAAGATCCCATTCATGGGAATGAAAAGAAAGGTGATACGTTTTGGAAAGAGGTTACTGATGAGTTCAACAGAAAAGGGAATGGGAAGCGTACAAGGGAAATAAATCAATTGAAGGTTCATTGGTCACGCCTCAAATCATCGATTGGAGAATTCAATGATTACTGGACTAAGGTAACTCAAATGAATACAAGCGGATATGACGATGACATGCTGGAGAAGGAGGCACAACAGATGTATGCAAATACATTTGGAAAGCCTTTTGCACTTGTGCATTGGTGGAAGATACTGAGAAAAGAGCCCAAGTGGTGTGCAATGATTGAGAAGGACAAAAACAAGGCTGAAGTGGTTGATATTCCAGATGAACAAAAGCGTCCCATTGGTAGAGAAGCAGCACAAGCCGAGCGCAATGGAAAACGCAAGAAGGACAGTATGTCAGAAGGAATTGTCATCCTAGGGGACAATATTGAAAAAATTATCAAAGTGACGCAAGATCGGAAGCTGGAGCGTGAGAAGGTCACTGAAGCACAGATTCACATTTCAAACGTAAATTTGAAGGCAGCAGAACAGCAAAAAGAAGCAAAGATGTTTGAGGTATACAATTCCCTGCTCACTCAAGATACAAGTAACATGTCTGAAGAACAGAAGGCTCGCCGAGACAAGGCATTACAAAAGCTGGAGGAAAAGTTATTTGCTGACTAAGGTTAGATATCTAATCTAATCTGAGCTGCACTATTATTTATAATAATTAAAGAATGCTGCAATATTTAGTTATATTGTCTGTATATCTGTGCTGCACTATGCAGTCAGCTGCATATCACGAATTTGTCAAATCTGAGCTGCATATCTGTGAATGGTGCAATATTTAGTTATATTAATTACCCAGTGTGAATGATGTATTGCTGTCAGTTTCACATATAGTATGAATGCTGCACTATGCAGTCAGTTTCACATGCAGTGTGAATGCTGCACTAGGCAGTCAGTTTCACATGCAGTGGGCGCCTATTTATGCAGAGTTTAGCCATCTCTCTACTCCTCTCAGAAACTCATTCCCTCTTTTCTCATACGAAGACCTCCTCCCTTTTATCTTTACTGTTTCTCTCTTCTTCAAAGATGTCTGAGCAAAATACTGATGGAAGTCAAGTTCCAGTGAACTTGTTGGATGAGTTCCTGGCTGAGGATGAGATCATAGATGATCTTCTCACTGAAGCCACGGTGGTAGTACAGTCCACTATAGAAGGTCTTCAAAACGAGGCTTCTGACCATCGACATCATCCGAGGAAGCACATCAAGAGGCCACGAGAGGAAGCACATCAGCAACTAGTGAATGATTACTTTTCAGAAAATCCTCTTTACCCTTCCAAAATTTTTCGTCGAAGATTTCGTATGTCTAGGCCACTTTTTCTTCGCATCGTTGAGGCATTAGGCCAGTGGTCAGTGTATTTCACACAAAGGGTGGATGCTGTTAATCGGAAAGGACTCAGTCCACTGCAAAAGTGTACTGCAGCTATTCGCCAGTTGGCTACTGGTAGTGGCGCAGATGAACTAGATGAATATCTGAAGATAGGAGAGACTACAGCAATGGAGGCAATGAAGAATTTTGTCAAAGGTCTTCAAGATGTGTTTGGTGAGAGGTATCTTAGGCGCCCCACCATGGAAGATACCGAACGGCTTCTCCAACTTGGTGAGAAACGTGGTTTTCCTGGAATGTTCGGCAGCATTGACTGCATGCACTGGCATTGGGAAAGATGCCCAGTAGCATGGAAGGGTCAGTTCACTCGTGGAGATCAGAAAGTGCCAACCCTGATTCTTGAGGCTGTGGCATCGCATGATCTTTGGATTTGGCATGCATTTTTTGGAGCAGCGGGTTCCAACAATGATATCAATGTATTGAACCAATCTACTGTATTTATCAAGGAGCTCAAAGGACAAGCTCCTAGAGTCCAGTACATGGTAAATGGGAATCAATACAATACTGGGTATTTTCTTGCTGATGGAATCTACCCTGAATGGGCAGTGTTTGTTAAGTCAATACGACTCCCAAACACTGAAAAGGAGAAATTGTATGCAGATATGCAAGAAGGGGCAAGAAAAGATATCGAGAGAGCCTTTGGTGTATTGCAGCGAAGATTTTGCATCTTAAAACGACCAGCTCGTCTATATGATCGAGGTGTACTGCGAGATGTTGTTCTAGCTTGCATCATACTTCACAATATGATAGTTGAAGATGAGAAGGAAACCAGAATTATTGAAGAAGATTTAGATCTAAATGTGCCTCCTAGTTCATCAACCGTTCAGGAACCTGAGTTCTCTCCTGAACAGAACACACCATTTGATAGAGTTTTAGAAAAAGATATTTCTATCCGAGATCGAGCGGCTCATAACCGACTTAAGAAAGATTTGGTGGAACACATTTGGAATAAGTTTGGTGGTGCTGCACATAGAACTGGAAATTGAGAATCAGTAAATGTAATTATTTTATTTTTCTTGTAATTTATATATCTATGGTCCACTTGTAAATTTCTGAATGCTCATCGCCATATTTTTTAATCTCTGCAGGTTCCAATCTATTTACAGGTTCCCTAAAAAAAAATCTATTTGCAGGTTCCAGTCTGTTGTCTTCACAATGTAAGTTCTGAGAATCAAATCACTATGTTTTTCTCTTTTTTGGTAGCTACAGGGTGTTAGAACATGTGTTATTTTCTTTACTATGCAATTGTGATCCTCCAATATTTATCTACTGCATGTGTAAACCTGTTTGTCATGTCTGAACTACTTTCATTTGTACAGGGTGAAAGAATCAATGAAATCTATGGGTGCATCGTCAATTTGCCTCCAGTTACCTGCTTGTCATCGTCATTTGTAGCTTAGTTCTGTCATATTTCACCTCGAGTTAACATCTATTCAGTTATCTAAACTTTGCTATGTAGTGAACTTGGTTGAATGGTCATTTAAATTTATCAAGTGAACAATCGTACCTATCTGTGCTGAATGCATGTATTTTGTTTTGTGTTCAAGTGGCTACACACGTTTGTGTTACATACGATCCCACTATGTGGCTGGAATTAAATGCCTTGAATTTGCATTGGAAACGCTAGAGTGAAACACAGCATTGAGAAGGTCTGTTTCATTGTACGTTTCAACTTGTTTCATCTTCGTTTCAGCTGATGTGGCGTCTGGGAAACAGTGTAATGAAACACTGCATTGTGAATGGCC
